# Fitness consequences of cousin marriage: a life-history assessment in two populations

**DOI:** 10.1017/ehs.2022.55

**Published:** 2022-11-29

**Authors:** Arianna Dalzero, Cody T. Ross, Dieter Lukas

**Affiliations:** Department of Human Behavior, Ecology and Culture, Max Planck Institute for Evolutionary Anthropology, Deutscher Platz 6, 04103 Leipzig, Germany

**Keywords:** Mating systems, cousin marriage, life-history, demography, kinship

## Abstract

Cousin marriage, a spousal union between close kin, occurs at high frequencies in many parts of the world. The rates of cousin marriage in humans are concordant with empirical studies that challenge the traditionally held view that reproduction with kin is generally avoided in animals. Similarly, some theoretical models in animal behaviour show that inbreeding avoidance is more constrained than previously thought. Such studies highlight the importance of quantifying the costs and benefits of reproduction among close kin over the whole life-course. Here, we use genealogical data from two human populations with high frequencies of cousin marriage (the Dogon from Mali, and the Ancien Régime nobility from Europe) to estimate these potential costs and benefits. We compare age-specific fertility and survival curves, as well as the projected growth rates, of subpopulations of each marriage type. Fitness costs of cousin marriage are present in terms of reduced child survival (in both populations), while benefits exist as increased fertility for men (in the Dogon) and for women (in the Ancien Régime nobility). We also find some differences in the projected growth rates of lineages as a function of marriage type. Finally, we discuss the trade-offs that might shape marriage decisions in different ecological conditions.

## Graphical abstract

**Figure:**
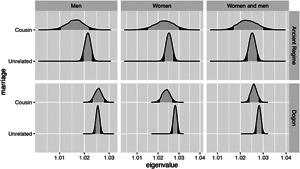

**Social media summary:** Survival and fertility benefits and costs of marrying a cousin, over the life-course of men and women in the Dogon and Ancien Régime.

## 1. Introduction

Cousin marriage, a spousal union of individuals who share one or more grandparents, is practised by more than 10% of the world's population, and was even more widespread before the demographic transition (Bittles & Black, 2010). Various medical studies have shown that reproduction among close kin can lead to potential reductions in fitness – often referred to in the existing literature as inbreeding costs – including both lower fertility and lower offspring survival rates (Bittles & Neel, 1994). However, theoretical work has shown that the conditions under which one should expect inbreeding avoidance are stricter than what is often assumed (Bateson, 1978; Kokko & Ots, 2006). Moreover, recent empirical analyses have revealed evidence of inbreeding tolerance, and even found some evidence of preferences for inbreeding in many animal species (Townsend et al., 2019; Nichols et al., 2014), challenging the widely held view that animals avoid mating with kin (de Boer et al., 2021; Pike et al., 2021). These studies highlight that the costs and benefits of reproductive strategies need to be considered jointly. Both costs and benefits are likely to depend on local socio-ecological conditions. Here, we draw on population projection models to quantify the life-history costs and benefits of cousin marriage, and we speculate on how different social and ecological conditions might shape the mating strategies that individuals deploy.

Several non-mutually exclusive hypotheses have been proposed to explain the potential fitness costs and benefits of cousin marriage. They provide various explanations for how cousin marriage, and a potential preference for it, might be maintained in a given population (Bailey et al., 2014; Shenk et al., 2016). For an overview of these hypotheses, and the predicted life history costs and benefits associated with each, see Table 1. Below, we provide a summary of the literature:

1. *The inbreeding cost hypothesis –* when individuals reproduce with relatives, they often suffer a reduction in fertility, and the survival of their offspring might be reduced – a phenomenon known as inbreeding depression (Pike et al., 2021; Charlesworth & Willis, 2009). Reproduction among close kin increases the chances that offspring are homozygous for deleterious recessive alleles. Costs of inbreeding have been documented among many human and non-human populations. For example, in the UK, the increased homozygosity among children born to cousins has been estimated to decrease their chances of having children by up to 55% (Clark et al., 2019). This mechanism is expected to reduce the frequency of cousin marriage, unless some other forces offset these costs.
2. *The genetic/environmental rescue hypothesis* – in populations where inbreeding is practised, demographic or selective processes might rapidly lead to a reduction in the frequency of deleterious alleles (Charlesworth & Willis, 2009; Hedrick & Garcia-Dorado, 2016). Alternatively, inbreeding costs are expected to be reduced or absent under benign circumstances, either if the whole population does not tend to experience harsh conditions (Kokko & Ots, 2006) or if related partners have differential access to resources (Ihle et al., 2017).
3. *The mate scarcity hypothesis* – cousin marriage might increase marriage opportunities (and therefore fertility) for individuals living in societies in which suitable partners are limited. In such cases, individuals choosing cousins as partners, and foregoing the costs of migration to find a mate, might benefit relative to individuals that spend more time on the mating market (Hoben et al., 2016; Cavalli-Sforza et al., 2004). Marriage opportunities can also be increased through marriage exchanges (Chagnon et al., 2017).
4. *The kin benefits hypothesis* – in certain circumstances, marriages between cousins might yield higher average reproductive success than marriages between unrelated individuals, if they provide access to kin-based wealth and alliances (Lévi-Strauss, 1971; Borgerhoff Mulder et al., 2009; Johow et al., 2019). The benefits of kin marriage might include: enhanced resource defence, increased access to inheritance and the prevention of resource dilution. For example, in some societies, marriage to a cousin could be the only way for women to avoid dowry payments or receive inheritance (Johow et al., 2019; Bahrami-Rad, 2021). Cousin marriage might also improve cooperation with in-laws (Willführ et al., 2018), enhance intensive kinship networks (Shenk et al., 2016) and promote nepotism (Akbari et al., 2019).
5. *The immune-response benefits hypothesis* – in areas with high pathogen prevalence, offspring of consanguineous parents might have higher survival rates, because reproduction among kin might preserve co-adapted gene complexes that control immune responses to pathogens (Hoben et al., 2016).

**Table 1.** Hypotheses and predictions (from research literature) for costs and benefits of cousin marriage

Hypothesis	Description	Predictions
1. Inbreeding costs	Fitness costs to accumulation of deleterious alleles	Lower fertility for cousin-married than unrelated-married individuals. Lower child survival for cousin-born than unrelated-born individuals
2. Genetic/environmental rescue	No inbreeding costs, owing to genetic rescue	Same fertility for cousin-married and unrelated-married individuals. Same survival for cousin-born and unrelated-born individuals
3. Mate scarcity	More opportunities in societies with limited partners	Lower age at first reproduction for cousin-married than for unrelated-married individuals
4. Kin benefits	Access to wealth, inheritance and alliances within families	Higher survival for cousin-born than for unrelated-born individuals. And/or, higher survival and fertility, and lower age at first reproduction for cousin-married than for unrelated-married individuals
5. Immune response benefits	Preserving co-adapted gene complexes that control immune response to pathogens	Higher survival for cousin-born than for unrelated-born individuals

The power of a specific hypothesis to explain the occurrence of cousin marriage in a specific society depends on the local socio-ecological circumstances. A combination of any of these hypotheses might apply in a given population. Costs and benefits might interplay in different ways, leading to trade-offs that can be expressed at an individual, family or population level. The trade-offs between costs and benefits indicate whether cousin marriage could be considered adaptive in a given population (Bolund, 2020).

Cousin marriage might entail different combinations of costs and benefits. First, there might be no detectable costs or benefits of cousin marriage, e.g. if past or current conditions remove the fitness consequences of this strategy (Kokko & Ots, 2006). Second, costs might outweigh benefits in families practising cousin marriage, e.g. if the cultural tradition is continued for reasons other than fitness maximization (Shenk et al., 2016). Third, any potential costs might be balanced by potential benefits in families practising cousin marriage, such that cousin marriage represents a viable mating strategy, which individuals might choose according to their personal circumstances (e.g. depending on their access to heritable wealth; Johow et al., 2019). Fourth, the benefits of cousin marriage might outweigh the costs, leading the majority of individuals to seek cousin marriages; in such cases, the frequency of cousin marriage in a population would be limited by the availability of suitable cousins as marriage partners (Givens & Hirschman, 1994). For example, Helgason et al. (2008) found a positive association between kinship and fertility, with a peak for third to fourth degree cousins, for couples born between 1800 and 1965 in the Icelandic population. Fifth, there might be parent–offspring conflicts when cousin marriages are arranged by parents. This can lead to direct costs for (some of) the children who marry cousins and benefits for their parents who – thanks to marriage exchanges – are more likely to find partners for all of their children, and therefore have more grandchildren (Chagnon et al., 2017). Sixth, same-sex siblings might compete over limited resources, such that cousin marriage might allow one sibling to have preferential access to material wealth and to exclude other same-sex siblings from inheritance (Johow et al., 2019). This could then lead to the exclusion of such siblings from the marriage market as well, especially in populations where material resources are essential for marriage (Hrdy & Judge, 1993). Seventh, cousin marriage might cause individuals of one sex to benefit, while individuals of the other sex face fitness costs (Waser et al., 1986). We follow the definition of Kokko and Jennions (2014), who state that sexual conflict occurs if the relative fitness of individuals of sex *A* is increased through the use of a strategy that impacts the behaviour of sex *B* at a fitness cost to sex *B*. In the context of cousin marriage, a sexual conflict could, for example, occur if men make agreements with their extended family to arrange the marriages of their sisters or daughters in exchange for additional marriage opportunities for themselves (Chagnon et al., 2017). In this scenario, men might stand to gain fitness benefits for themselves while simultaneously burdening their female kin with fitness costs – costs that they would not face if they had a free choice of partners.

### 1.1. Approach

A thorough evaluation of whether the proposed benefits of cousin marriage are sufficient to make the practice adaptive in the long-term is still lacking. Previous studies have been somewhat limited by focusing on one hypothesis at a time and testing each hypothesis with specific – but limited – fitness proxies, or by focusing on short-term contexts only (Bailey et al., 2014; Chagnon et al., 2017). Simple proxies of reproductive success, such as the number of offspring or grand-offspring, can potentially indicate differences in fitness, but they cannot be used to infer long-term fitness consequences, as they ignore variation in key aspects of fitness, such as survival trajectories and fertility scheduling (Coulson et al., 2006). These short-term fitness proxies are particularly limited in humans, because of our overlapping generations and flexible reproductive scheduling (Westneat et al., 2010). In order to understand the long-term consequences of mating strategies, such as cousin marriage, we need to consider the costs and benefits of the strategy across all key phases of life-history, and then determine projected long-term population dynamics (Kokko & Ots, 2006; Szulkin et al., 2013). Here, we use demographic methods and age-structured statistical models, which allow us to jointly investigate the effects of cousin marriage on both survival and fertility across the life course, and thus determine long-term population dynamics (Caswell, 2000). This approach is readily applicable to data on cousin marriage in humans, given the availability of demographic and genealogical records from many populations. We expect the potential costs and benefits of cousin marriage to be population specific, because local conditions – such as resource availability or mating system – are likely to shape life-history strategies.

We focus here on two populations in which cousin-marriage is practised at rates higher than what would be expected if individuals chose partners randomly. The first population studied here is the Dogon from Mali. This population is endogamous, and the proportion of marriages among first cousins (15%) is more than twice as large as what would be predicted from the proportion of marriageable first cousins in each age group (Brown, 1993; Hajnal, 1963). Matrilateral cross-cousin marriage (i.e. marriage between a focal male and his mother's brother's daughter) is the traditional – and still most common – form of cousin marriage. However, patrilateral parallel cousin marriage (i.e. marriage between a focal male and his father's brother's daughter), and other forms of cousin marriage, began to increase in the twentieth century, after large-scale conversions to Islam occurred (Cazes, 2006). Cross-cousin marriage among the Dogon is thought to be driven by the traditional matrilineal system of land inheritance (i.e. where inheritance goes to the sons of the sisters of a focal man) and by matrimonial exchanges that enhance cooperation within lineages (Strassmann and Kurapati, 2016). Parallel cousin marriage, in contrast, is thought to be driven by the more recent arrival of an Islamic patrilineal system of inheritance (Cazes, 2006). On the basis of these previous studies, we would expect that cousin marriage in this population is potentially beneficial for males, with sons trying to secure inheritance and keep it in the family by marrying their cousins.

The second population studied here comprises members of the Ancien Régime Western European nobility. This population is endogamous too, even if the population is not limited to a small geographic region, because marriages were arranged only within nobility (Hurwich, 1998). Cousin marriage was present among the nobility in early modern Europe, especially in royal dynasties, such as the Habsburgs. Starting from 500 AD, the Roman Catholic Church banned cousin marriage. However, papal dispensations were granted especially to nobility; after the Reformation, Protestant denominations accepted first-cousin marriage (Henrich, 2020). Cousin marriage among the European nobility has been seen as an inward political alliance, and a tool for territorial control, within dynasties. Most cousin marriages among royal dynasties were among cross-cousins, reinforcing alliances made in earlier generations, with cases of parallel cousin marriage sometimes occurring when daughters were the only living heirs (Fleming, 1973). Additionally, cousin marriage among this limited set of individuals might provide more secure prospects of marriage in contexts where the local pool of unrelated prospective spouses was small (Bouchard, 1981). In the context of European nobility, cousin marriage might have also allowed parents to avoid paying dowry for their children, and to keep their daughters’ wealth in the family (Do et al., 2013; Bahrami-Rad, 2021). An alternative strategy to cousin marriage in this population involved the arrangement of marriage between unrelated individuals for the purposes of political alliance formation and territory acquisition. Research indicates that inbreeding at the level of first cousin had an adverse effect (of about 12.3–17.8%) on offspring survival in the Spanish branch of the Habsburg dynasty (Alvarez et al., 2009). As such, we would expect that cousin marriage should deliver some kind of benefit to offset these costs.

The two populations described here have very different socio-ecological contexts (e.g. social and mating systems). We therefore predict that men and women in these two societies will be affected differently by cousin marriage. In typical cases of sexual conflict, the deviation from optimal fitness that one sex imposes on the other is context dependent (Kokko and Jennions, 2014). For both populations, we use genealogical data to compare age-specific life-history estimates among individuals who are either in cousin marriages or in marriages to unrelated individuals. We test the aforementioned hypotheses on the basis of the implications that they have for life-history outcomes (Table 1). Specifically, we study how the probability of child survival up to reproductive age, adult survival during reproductive ages, age at first reproduction and overall fertility covary with the practice of cousin marriage. We then use these age-specific probabilities of survival and reproduction to perform population projections. This allows us to estimate the projected growth rates of lineages practising versus not practising cousin marriage. We use these demographic measures to investigate the potential fitness trade-offs entailed in cousin marriage in a given population. The population growth rate is frequently used as the fitness measure of choice for age-structured populations (Jones, 2006). If the population growth rate of lineages practising cousin-marriage is similar to, or higher than, the population growth rate of lineages not practising cousin marriage, this would indicate that the costs associated with cousin marriage are compensated for, or balanced by, benefits. In contrast, if the population growth rate of lineages practising cousin-marriage is lower than the population growth rate of lineages not practising cousin marriage, this would indicate that the costs associated with cousin marriage outweigh the benefits. Additionally, asymmetries between sexes in age-specific probabilities of survival and reproduction in lineages practising cousin marriage might indicate sexual conflict connected to cousin marriage. The sensitivity of the population growth rate to changes in survival and fertility represents the force of selection on the phenotype (Jones, 2006). Accordingly, we also conduct a sensitivity analysis in each population.

## 2. Methods

### 2.1. Data sources and ethnographic overview

#### 2.1.1. Dogon from Mali

The first long-term demographic and genealogical dataset used in this study comes from a Dogon population in Mali. These data were collected by Marie-Helene Cazes (2016) and obtained via the Kinsources archive: https://www.kinsources.net/kidarep/dataset-186-dogon-boni.xhtml. This population is composed of isolated groups of Dogon, living in the semi-arid climate of the southern Sahel in Mali, around the district of Boni. The population is spread across 15 villages and four mountain settlements. The data were collected from 1975 to 1987, for the purpose of demographic and genetic research, and contain records spanning a period from 1920 to 1987. This dataset includes information about 11,294 individuals, and 4,594 families, over 17 generations. It is an ‘exhaustive census’ of the population; all living individuals in the population were sampled and information about their family structure was recorded (Brown, 1993). Around 15% of all marriages (and 19.5% of first marriages) in this sample are between first cousins (Brown, 1993). About 45% of men in cousin-marriages were married to their mother's brother's daughter (matrilateral cross-cousin), 28% to their father's brother's daughter (patrilateral parallel cousin), 20% to their father's sister's daughter (patrilateral cross-cousin) and 7% to their mother's sister's daughter (matrilateral parallel cousin). The majority of marriages in this population are monogamous; however, non-sororal polygynous marriages are allowed in Dogon culture and men have an average of 1.3 simultaneous wives (Brown, 1993). According to Strassmann (2017), higher mortality for men than for women, urban migration and age differences between spouses (men marry at a later age) lead to a female-biased operational sex ratio that promotes polygyny among the Dogon. Successive remarriages are also common, with men having an average of 2.7 total partners by the time they reach 45 years of age (Brown, 1993). Failure to marry appears very rare in this population (with only 1% of men and 0% of women remaining unmarried to age 40) (Brown, 1993). Total life-time fertility is high (7.2 children per woman on average) and dependent on spousal number, especially for men. Mortality, especially in infancy, is high as well, with up to 40% of children not reaching the age of five in some periods (Brown, 1993). Men have a 10% higher mortality rate than women during pre-reproductive ages (Brown, 1993).

#### 2.1.2. Ancien Régime from Western Europe

The second dataset used in this study is a genealogy of European nobility from Western Europe (France, Italy, Germany and the UK) between the 16th and the 18th centuries (Public Domain, 2010), and was also provided by the Kinsources archive: https://www.kinsources.net/kidarep/dataset-53-ancien-regime.xhtml. The genealogy contains all cognatic kin of Louis XVI and Marie Antoinette up to the sixth degree. The data have been drawn from a variety of printed and online sources. The dataset includes information on about 5891 individuals, and 2399 families, across 11 generations. About 4.1% of marriages in the sample are between first cousins (2.2% cross-cousin marriages, 1.92% parallel cousin). Only 6% of individuals in this dataset who survived to adulthood do not appear as married. In these data, 54% of marriages produced children, at an average rate of three children per couple. Monogamy is predominant: 94% of married women and 82% of married men are married only once (Public Domain, 2010).

### 2.2. Data analyses

We use the same statistical methods to analyse data from both Dogon and Ancien Régime populations. We estimate age-specific probabilities of survival and reproduction for subpopulations of individuals (e.g. those in cousin marriages and those in marriages with unrelated partners). Using these age-specific estimates, we build age-structured population projection models (Leslie, 1945). We observe and compare the demographic characteristics of different subpopulations, and analyse the posterior distributions of the predicted long-term growth rates. We perform the analyses using R (R Core Team, 2020) and Stan (Stan Development Team, 2021; Carpenter et al., 2017). All code needed to reproduce our analyses will be maintained at: https://doi.org/10.17605/OSF.IO/DGUF5.

### 2.3. Data preparation and relatedness analyses

The Dogon and Ancien Régime databases each contain two data files. The first file contains information at the individual level, and includes a unique ID, gender, birth date and death date, for each individual. The second file contains information at the family level, and includes a household ID, mother ID, father ID and child IDs, for each family. We create 12 subpopulations of individuals: (1) women married to cousins; (2) women married to unrelated partners; (3) women born to cousins; (4) women born to unrelated parents; (5) men married to cousins; (6) men married to unrelated partners; (7) men born to cousins; (8) men born to unrelated parents; (9) individuals, men or women, married to cousins; (10) individuals married to unrelated partners; (11) individuals born to cousins; and (12) individuals born to unrelated parents. We test if individuals are in cousin marriages by assessing if individuals who are partners have at least one grandparent in common. Individuals who are classified as ‘born to cousins’ have parents with at least one grandparent in common. Likewise, individuals who are classified as ‘married to cousins’ are those individuals who, in their lifetimes, have had at least one partner with whom they have shared a grandparent. We excluded records from individuals for whom all eight grandparents are unknown, because we could not accurately classify their relatedness to other individuals. For the Dogon, we conducted a robustness check by repeating the analyses and including only individuals for whom all eight grandparents are known (the results of this robustness check are consistent with those of the main analyses). All of the ‘married’ groups contain individuals who are married. The ‘born to’ groups contain individuals who might be unmarried, married to cousins, or married to unrelated individuals. For each individual, we note the age of death, or, in the case of the Dogon, the age of censoring, if they were still alive at the end of data collection. For each parent, we assign an age-at-birth for each of their offspring, by subtracting the offspring's birth date from the parent's birth date. In the Ancien Régime database, we corrected 60 inconsistent dates of birth and death, by cross-checking these data with other sources of information. In the Ancien Régime database, we found around 1000 missing birth and death dates, and found that they belonged mainly to individuals who had died in infancy. These missing records might bias estimates of age-specific survival and fertility, but this bias should not be dependent on the type of marriage an individual is in. We discuss the possible inferential limitations imposed on our study by these missing data in the Discussion section.

### 2.4. Survival and fertility estimation

To estimate age-specific survival and fertility curves, we built Bernoulli models in Stan (Carpenter et al., 2017), using a Gaussian Process approach to model random effects on each age category. This approach allows us to estimate the functions that link survival and age, as well as fertility and age, without knowing *a priori* what shape these functions have.

If individual *i* is a woman, we define *Y*_[*i*,*a*]_ to be a binary variable indicating if she produced a daughter at age *a*. Likewise, if individual *i* is a man, we define *Y*_[*i*,*a*]_ to be a binary variable indicating if he produced a son at age *a*. We then model these fertility outcomes as:1
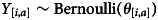
where *θ*_[*i*,*a*]_ is the probability that individual *i* produces an offspring at age *a*. We model *θ*_[*i*,*a*]_ as a function of random effects:2
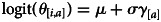
where3
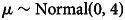


*σ* is a scalar, and *γ* comes from a multivariate normal distribution:4
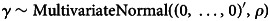


We then model the correlation matrix, *ρ*, using a Gaussian process approach:5
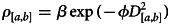
where *D*_[*a*,*b*]_ is the normalized distance between age categories *a* and *b*, *β* is the maximal correlation (when the distance between age categories is 0) and finally *ϕ* is a parameter which controls the rate of decay of correlation with distance between age categories.

To complete the model, we put weak priors on the top-level parameters:6
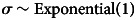
7
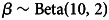
8
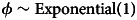
We fit this model independently to different subsets of data (e.g. males in cousin marriages). The survival model is of the same form as the fertility model, but the variable *Y*_[*i*,*a*]_ is replaced with the variable *S*_[*i*,*a*]_, which defines if individual *i* survived age *a*.

From each model, we extract the posterior distributions of all parameters and use them to reconstruct all relevant survival and fertility curves. Child survival is defined as the cumulative survival probability up to the earliest age of observed reproduction (i.e. age 12). Adult survival is defined as the cumulative survival probability across reproductive ages in each population (i.e. ages 12–60). Overall fertility is defined as the sum of age-specific fertility across reproductive ages. The age at first reproduction is defined to be the first age when the expected number of births exceeds 0.5.

### 2.5. Population projections

We use the estimated age-specific survival probabilities and fertility rates to construct deterministic Leslie matrices, which are used to conduct population projections (Leslie, 1945). For the one-sex analyses, we construct the Leslie matrices as arrays containing age-specific fertility rates in the top row, age-specific survival probabilities on the subdiagonal, and zeros elsewhere (Jones, 2006; Caswell, 2000). For the two-sex analyses, we assemble the Leslie matrices described above to build a two-sex matrix, which is composed of four blocks (see details in Gerber and White, 2014; Abaitey and Oduro, 2017). We first build Leslie matrices where we define parameters on the basis of birth, to explore projected population growth rates for the various subpopulations under investigation here. These matrices use the survival estimates of, for example, women *born* to cousins, and the fertility estimates of women *born* to cousins. Additionally, we build Leslie matrices where we define parameters on the basis of both birth and marriage. These Leslie matrices use, for example, survival estimates for individuals whose parents are cousins and fertility estimates from individuals who are married to cousins. These mixed matrices are aimed at creating more ‘realistic’ subpopulations and imply the assumption that cousin marriage is heritable in the lineage and that individuals whose parents are cousins marry cousins.

For each of the Leslie matrices, at each MCMC sample, we calculate the dominant eigenvalue of the matrix. We then visualize the posterior distribution of the dominant eigenvalue, in order to represent our uncertainty in these estimates. The dominant eigenvalue represents the asymptotic growth rate of the population, i.e. the rate at which the population grows exponentially after reaching the stable age distribution (Jones, 2006). Using the popdemo package in R (Stott et al., 2012), we estimate the distribution of eigenvalue sensitivities for survival and fertility. These are linear estimates of the change in population growth rate given a perturbation in survival or fertility (i.e. they represent the partial derivative of the population growth rate with respect to different input parameters; Jones, 2006).

### 2.6. Results computation

For each trait of interest, we compute the contrast (i.e. the difference) between the posterior probability distributions of the relevant subpopulations. Specifically, we use the rethinking package in R (McElreath, 2020) to compute and analyse the mean and the compatibility intervals of the posterior distributions (89% PI), as well as the fraction of the posterior above and below zero. We define *p* to be the integral between zero and infinity of the distribution of the contrast. As such, *p* = 0.95 indicates that 95% of the posterior density is above zero, while *p* = 0.5 indicates that exactly half of the posterior density is above zero. Values of *p* close to one or zero (i.e. *p* > 0.95 or *p* < 0.05) are indicative of stronger certainty for a non-zero effect, while values near 0.5 indicate that the compared subpopulations have similar estimates. We perform comparisons as a function of the marriage type of an individual, or of the marriage type of an individual's parents. Additional comparisons of fertility and age at first reproduction between born and married subpopulations (e.g. women born to cousins vs. women married to cousins) are presented in the Supplementary Materials. All comparisons are between same-sex subpopulations.

#### 3. Results

In the following subsections, we first describe the results for survival and fertility functions, and then investigate projected growth rates and sensitivities. In Tables 2 (Dogon) and 3 (Ancien Régime), we present a summary of the results of the estimated mean and the fraction of the posterior above zero (*p*). Full results, including a larger set of analyses, are provided in the Supplementary Materials, Tables S1–S32. Outcomes not presented in the figures in the main manuscript are provided in the Supplementary Materials, Figures S1–S5.

**Table 2.** Dogon life-history comparisons. Estimated mean contrast, and the fraction of posterior density above 0 (*p*), for each category of interest. Values close to *p* = 0 or *p* = 1 indicate strong evidence of a directional effect as indicated by the sign of the mean contrast. Values near 0.5 indicate no reliable difference.

	Child survival	Adult survival	Fertility	Age at first reproduction	Growth rate
Women cousin-born vs. women unrelated-born	Mean = −0.06, *p* = 0.00	Mean = −0.1, *p* = 0.01	Mean = −0.17, *p* = 0.16	Mean = 0.15, *p*= 0.22	Mean = −0.0078, *p* = 0.00
Men cousin-born vs. men unrelated-born	Mean = −0.04, *p* = 0.02	Mean = 0.02, *p* = 0.59	Mean = 0.28, *p* = 0.88	Mean = 0.6, *p*= 0.57	Mean = −0.0012, *p* = 0.26
Women cousin-married vs. women unrelated-married		Mean = −0.01, *p* = 0.37	Mean = 0, *p* = 0.48	Mean = −0.88, *p* = 0.00	
Men cousin-married vs. men unrelated-married		Mean = 0.04, *p* = 0.75	Mean = 0.39, *p* = 0.99	Mean = −0.95, *p* = 0.00	
Women cousin-born and cousin-married vs. women unrelated-born and unrelated-married					Mean = −0.043, *p* = 0.02
Men cousin-born and cousin-married vs. men unrelated-born and unrelated-married					Mean = 0.0002, *p* = 0.55
Women and men cousin-born and cousin-married vs. women and men unrelated-bornand unrelated-married					Mean = −0.0022, *p* = 0.08

**Table 3.** Ancien Régime life-history comparisons. Estimated mean contrast, and the fraction of posterior density above 0 (*p*), for each category of interest. Values close to *p* = 0 or *p* = 1 indicate strong evidence of a directional effect as indicated by the sign of the mean contrast. Values near 0.5 indicate no reliable difference.

	Child survival	Adult survival	Fertility	Age at first reproduction	Growth rate
Women cousin-born vs. women unrelated-born	Mean = −0.01, *p* = 0.42	Mean = −0.07, *p* = 0.12	Mean = −0.06, *p* = 0.41	Mean = −1.69, *p*= 0.09	Mean = −0.0025, *p* = 0.34
Men cousin-born vs. men unrelated-born	Mean = −0.06, *p* = 0.09	Mean = 0.03, *p* = 0.70	Mean = 0.18, *p* = 0.70	Mean = −3.58, *p*= 0.01	Mean = −0.0002, *p* = 0.49
Women and men cousin-born vs. women and men unrelated-born					Mean = −0.0012, *p* = 0.40
Women cousin-married vs. women unrelated-married		Mean = −0.01, *p* = 0.42	Mean = −0.02, *p* = 0.46	Mean = −1.17, *p* = 0.08	
Men cousin-married vs. men unrelated-married		Mean = −0.01, *p* = 0.44	Mean = −0.21, *p* = 0.20	Mean = 0.17, *p* = 0.34	
Women cousin-born and cousin-married vs. women unrelated-born and unrelated-married					Mean = −0.023, *p* = 0.32
Men cousin-born and cousin-married vs. men unrelated-born and unrelated-married					Mean = −0.0051, *p* = 0.09
Women and men cousin-born and cousin-married vs. women and men unrelated-bornand unrelated-married					Mean = −0.0019, *p* = 0.32

### 3.1. Contrasts in child and adult survival

#### 3.1.1. Dogon

**Figure 1.**
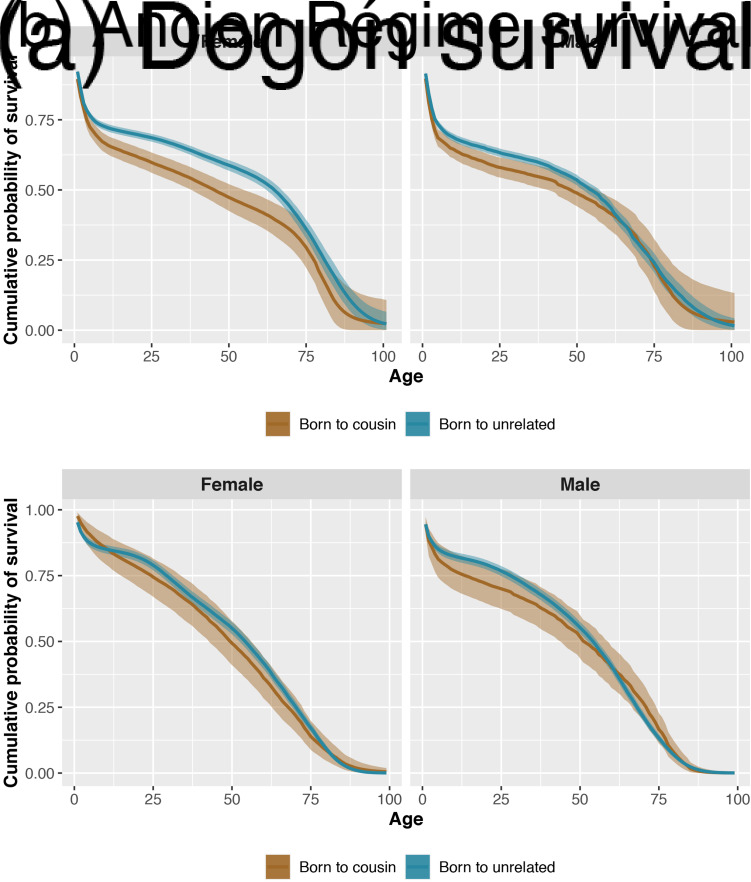
Posterior probabilities of cumulative survival (*y*-axis) for each age of life (*x*-axis) for female (left panels) and male (right panels) Dogon (frame a) and Ancien Régime (frame b) individuals. Estimates for the offspring of cousins are coloured brown and estimates for the offspring of unrelated parents are coloured blue. The bands represent 90% compatibility intervals. Fitness costs in survival, especially in early ages, are particularly visible in female Dogon individuals.

Individuals born to cousins are less likely to survive to reproductive age (i.e. 12 years old) compared with individuals born to unrelated parents. This effect is slightly stronger for female children (where the expected difference in survival probability is −0.06, *p* = 0.00), than male children (where the expected difference in survival probability is −0.04, *p* = 0.02; see Figure 1). Women born to cousins are also less likely to survive as adults (i.e. during their reproductive ages 12–60, where the expected difference in survival probability is −0.10, *p* = 0.01). Men married to cousins are slightly more likely to survive as adults than men married to unrelated partners (expected difference in survival probability is 0.04, *p* = 0.75). Women married to cousins have similar adult survival probabilities to women married to unrelated partners.

#### 3.1.2. Ancien Régime

Male individuals born to cousins are less likely to survive up to 12 years old compared with male individuals born to unrelated parents (i.e. the expected difference in survival probability is −0.06, *p* = 0.09). In contrast, female individuals born to cousins have the same child survival probability as female individuals born to unrelated parents. However, female individuals born to cousins have a lower survival probability as adults than female individuals born to unrelated parents (expected difference −0.07, *p* = 0.12; see Figure 1). Both women and men married to cousins have the same adult survival probabilities as their unrelated counterparts.

### 3.2. Contrasts in fertility: total fertility and age at first reproduction

#### 3.2.1. Dogon

**Figure 2.**
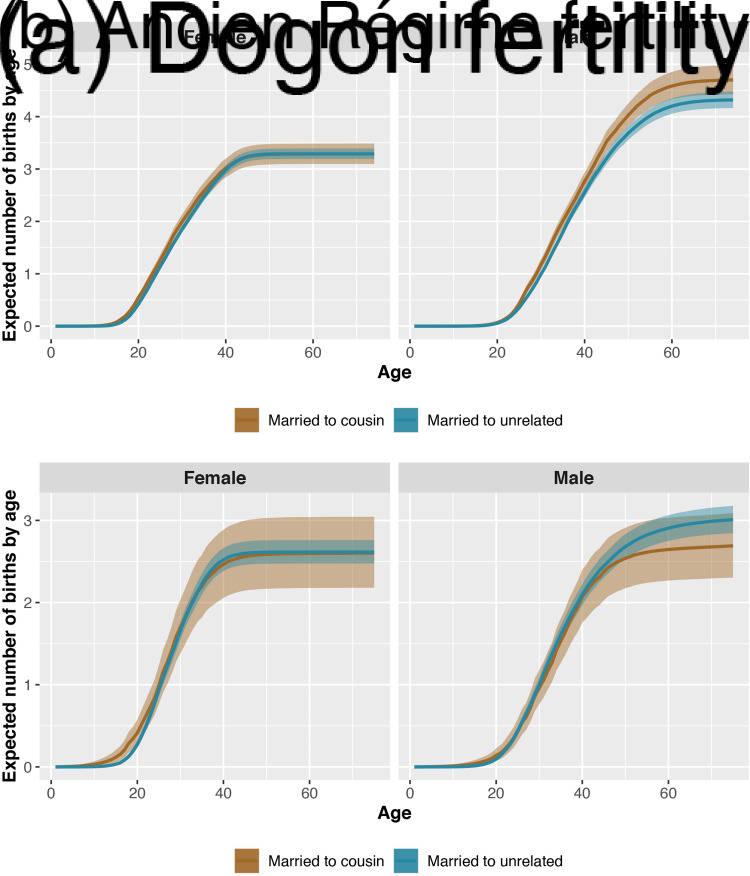
Expected number of children (*y*-axis) for each age of life (*x*-axis) for female (left panels) and male individuals (right panels) who are married to cousins (brown) and married to unrelated partners (blue). The bands represent 90% compatibility intervals. These curves reflect the expected number of same-sex children (daughters of women and sons of men) produced by individuals. Men's reproductive careers in the Dogon (frame a) begin later than women's, and their fertility is slightly higher because of polygyny (as estimates exclude unmarried individuals). In the Dogon, men married to cousins have slightly higher fertility than men married to unrelated partners. In the Ancien Régime (frame b), men married to cousins have slightly lower fertility than men married to unrelated partners.

Total fertility is slightly lower for women born to cousins than for women born to unrelated parents (expected difference −0.17, *p* = 0.16), but higher for men born to cousins than for men born to unrelated parents (expected difference 0.28, *p* = 0.88; see Figure S1). Total fertility is higher for men married to cousins than for men married to unrelated partners (expected difference 0.39, *p* = 0.99). We find no reliable differences in women's fertility as a function of marriage type (Figure 2). Both women and men born to cousins have the same age at first reproduction as women and men born to unrelated parents. Age at first reproduction is lower (i.e. earlier) both for men married to cousins compared with men married to unrelated partners (expected difference −0.95, *p* = 0.00), as well as for women married to cousins compared with women married to unrelated partners (expected difference −0.88, *p* = 0.00).

#### 3.2.2. Ancien Régime

Both women and men born to cousins have similar total fertility to their counterparts who are born to unrelated parents (Figure S2). However, total fertility is slightly lower for men married to cousins than for men married to unrelated partners (expected difference −0.21, *p* = 0.2; see Figure 2). We find no other differences in women's fertility as a function of marriage type. For both women and men, age at first reproduction is earlier for those born to cousins than for those born to unrelated parents (expected difference for women −1.69, *p* = 0.09; expected difference for men −3.58, *p* = 0.01). Age at first reproduction is also earlier for women married to cousins than for women married to unrelated partners (expected difference −1.17, *p* = 0.08). We find no differences in men's age at first reproduction as a function of marriage type.

### 3.3. Contrasts in growth rates and sensitivities

#### 3.3.1. Dogon

For the base model, we construct Leslie matrices using the survival estimates of, for example, women *born* to cousins and the fertility estimates of women *born* to cousins. The projected growth rate of such a hypothetical population of women practising cousin marriage is lower than the projected growth rate of a hypothetical population of women not practising it (expected difference −0.008, *p* = 0.0003). Similarly, such estimates are slightly lower for a hypothetical population of men practising cousin marriage than men not practising cousin marriage (expected difference −0.0012, *p* = 0.2620). For the two-sex model integrating both men and women, the growth rate is lower for the hypothetical population practising cousin marriage than for the hypothetical population not practising it (expected difference −0.0037, *p* = 0.0252; see Figure S3). The sensitivities of the population growth rate to changes in fertility are very slightly lower for the hypothetical subpopulation of men practising cousin marriage than for men not practising it. We find no differences in women's fertility sensitivities or in women's or men's survival sensitivities.

**Figure 3.**
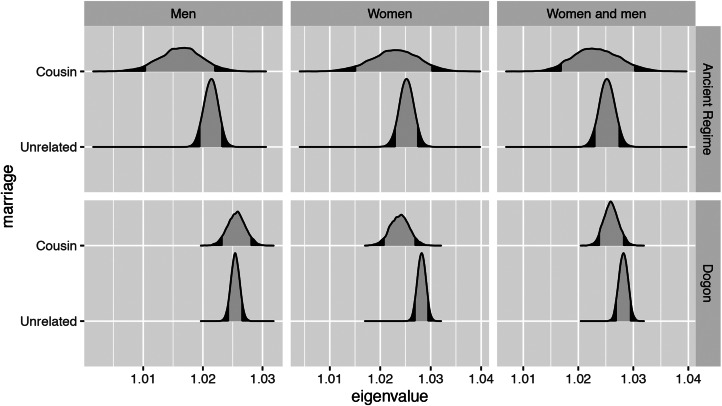
Projected growth rates are shown here using the density (*y*-axes) of the posterior distribution of the estimated dominant eigenvalue (*x*-axes). Estimates for the Ancien Régime appear in the top set of rows, and estimates for the Dogon appear in the bottom set of rows. Each estimate represents the projected growth rate of a hypothetical subpopulation of men (left column), women (central column) or men and women together (right column) from the Leslie matrices where we define parameters on the basis of both birth and marriage. Survival rates are from individuals who are born to cousins (or born to unrelated parents) and fertility rates are from individuals who are married to cousins (or married to unrelated partners). In the Dogon, the growth rate of a hypothetical population of women practising cousin marriage is slightly lower than the growth rate of a hypothetical population of women not practising it. For Dogon men in hypothetical lineages practising cousin marriage, there are no overall fitness consequences compared with men in hypothetical lineages not practising cousin marriage. In the Ancien Régime, hypothetical lineages of men practising cousin marriage have a lower growth rate than hypothetical lineages of men not practising it. However, no differences in lineage growth rate are observed for women.

**Figure 4.**
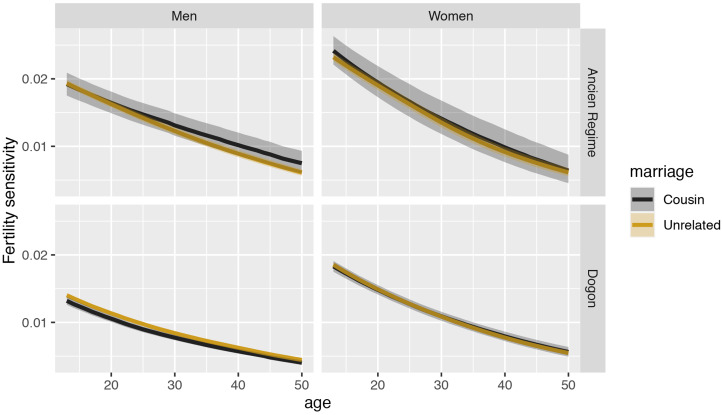
Sensitivity of the projected lineage growth rate to changes in fertility (on the *y*-axis) for each reproductive age (on the *x*-axis) for both Dogon (bottom row) and Ancien Régime (upper row) populations. Estimates for men are plotted in the left column and estimates for women are plotted in the right column. Estimates for those practising cousin marriage are plotted in black and estimates for those not practising it are plotted in yellow. The shaded regions represent the 90% compatibility intervals.

Additionally, we construct Leslie matrices where we define parameters on the basis of both birth and marriage (e.g. with survival estimates coming from individuals whose parents are cousins and fertility estimates coming from individuals who are married to cousins). The projected growth rate of such a hypothetical population of women practising cousin marriage is lower than the projected growth rate of a hypothetical population of women not practising it (expected difference −0.0043, *p* = 0.0163). The projected growth rate of such a hypothetical population of men practising cousin marriage is the same as the projected growth rate of a hypothetical population of men not practising it. The projected growth rate of a hypothetical population of women and men practising cousin marriage is lower than the projected growth rate of a hypothetical population of women and men not practising it (expected difference −0.0022, *p* = 0.0797; see Figure 3). The sensitivities of the population growth rate to changes in fertility from this set of Leslie matrices are very slightly lower for the hypothetical subpopulation of men practising cousin marriage than for men not practising it. We find no differences in the fertility sensitivities of women (Figure 4) and in the survival sensitivities of men (Figure S5). Women in lineages practising cousin marriage have slightly higher survival sensitivity in early ages (Figure S5).

#### 3.3.2. Ancien Régime

For the Ancien Régime population, in all the base models defining parameters on the basis of birth, the projected growth rates for a hypothetical population practising cousin marriage and a hypothetical population not practising it are similar (Figure S4). In this set of models, we find no differences in the fertility and survival sensitivities of the projected population growth rate.

In the set of models where we define parameters on the basis of both birth and marriage, the projected growth rate of a hypothetical population of men practising cousin marriage is lower than the projected growth rate of a population of men not practising it (expected difference −0.0051, *p* = 0.086). We find no differences in projected growth rates between hypothetical populations of women practising vs. not practising cousin marriage (Figure 3). In the two-sex model, we also find no differences. The sensitivities of the population growth rate to changes in fertility are slightly higher for the hypothetical subpopulation of men practising cousin marriage than for men not practising it. We find no differences in the fertility sensitivities of women (Figure 4), and we find no differences in the survival sensitivities of women and men (Figure S5).

## 4. Discussion

### 4.1. Overview

The application of demographic methods to data from the Dogon and Ancien Régime populations allows us to estimate the fitness costs and benefits associated with cousin marriage. The costs associated with cousin marriage are more visible in the Dogon, and are particularly striking in the reduced childhood survival probabilities for individuals born to cousin-married parents. These findings recapitulate the findings of a large number of studies suggesting that there are fitness costs when close kin reproduce, in line with the inbreeding costs hypothesis from biology (Bittles & Neel, 1994). In terms of fitness benefits, we find, in the Dogon, increased fertility and lower ages of first reproduction and, in the Ancien Régime nobility, lower age at first reproduction among individuals married to cousins. These findings are compatible with the idea that fitness benefits accrue through improved chances of marriage, as predicted by the *mate scarcity hypothesis*, and through improved access to resources, as predicted by the *kin benefits hypothesis*. Importantly, in both the Dogon and Ancien Régime populations, the costs of cousin marriage are predominantly carried by one sex, while the benefits are mainly gained by the other sex. Interestingly, the sex that benefits from cousin marriage differs across these populations. In each population, the allocation of costs and benefits between the sexes indicates potential sexual conflict. The expression of sexual conflict in these two populations seems to be dependent on the marriage system of each.

Because we observe evidence of fitness costs to cousin marriage, in terms of survival, while simultaneously failing to find large differences in projected growth rates, our results suggest that there may be trade-offs, such that fitness benefits in terms of fertility might be mitigated by increased mortality costs. However, the compensation of costs seems to be incomplete in the Dogon, as the projected growth rates of lineages practising cousin marriage appear to be slightly lower than the projected growth rates of lineages not practising cousin marriage.

### 4.2. Life-history trade-offs in cousin marriage

#### 4.2.1. Dogon

Among the Dogon, the life-history consequences associated with cousin marriage are limited, but detectable. It is notable that many Dogon individuals who are not married to first cousins are nevertheless married to slightly more distant relatives, owing to endogamy (Brown, 1993). Fitness costs appear to be mainly carried by women, while fitness benefits are mainly gained by men (consistent with the *sexual conflict hypothesis*).

The main fitness costs associated with cousin marriage in the Dogon are lower offspring survival and, to a lesser extent, lower fertility. We found that, for the Dogon, both sons and daughters born to cousins have lower chances to survive to reproductive age than sons and daughters born to unrelated parents. It is unlikely that these fitness costs are linked to lower socio-economic status of families practising cousin marriage, because we do observe fitness benefits that presumably reflect higher resource access in these families (see below) (Strassmann, 2017). A possible explanation for the overall reduced offspring survival in cousin-married families could be the biological consequences of mating with kin, which could also explain why women born to parents who are cousins have lower fertility than women born to unrelated parents. In addition, children born in families practising cousin marriage might also have higher mortality if they have more siblings, particularly in polygynous families, because, among the Dogon, an increase in the ratio of children to adults in a family appears to be associated with increased child mortality (Strassmann, 2017). Even though sons and daughters of cousins have roughly equal survival rates, the survival costs of cousin marriage appear most pronounced among female offspring of cousin-married couples. This is because daughters of unrelated parents show reliably higher rates of survival than sons of unrelated parents. The difference in survival between daughters and sons in families not practising cousin marriage (see also, Brown, 1993) seems not to be due to less childcare given to sons than to daughters (Strassmann, 2017), but potentially reflects the higher physiological growth costs and higher vulnerability of sons (Wells, 2000; Helle et al., 2002; Clutton-Brock et al., 1981). The sex-bias in survival might disappear in cousin-married families because offspring mortality reaches a ceiling.

As for the potential benefits of cousin marriage, men married to cousins show slightly higher fertility and earlier ages at first reproduction compared with men married to unrelated partners. The traditional matrilateral cross-cousin marriage system of the Dogon reinforces the traditional matrilineal system of inheritance, while the newer Islamic patrilateral parallel-cousin marriage system reinforces the recently imported patrilineal system of inheritance (Cazes, 2006). In Dogon society, the first marriage of each man is decided on by the head of the family and is preferentially arranged to a cousin, whereas the following wives are freely chosen by the husband (Cazes, 2006). The earlier age at first reproduction for cousin-married individuals (especially males) points to the possibility that cousin marriage eases the problem of finding a mate in this population (as predicted by the *mate scarcity hypothesis*). In addition, by arranging a marriage between his daughter and his sister's son, a man can indirectly pass on his wealth to his own grandchildren, without violating traditional inheritance norms. Cousin marriage can also establish family ties, which are an important factor in cooperation, land defence and cultivation among Dogon (Strassmann & Kurapati, 2016). Such access to resources might increase the reproductive success of men in multiple ways (as predicted by the *kin benefits hypothesis*). There could be an influence of increased resource access on the physiology of men and their children (Borgerhoff Mulder & Beheim, 2011). In addition, young Dogon men might have to migrate for periods of up to 2–3 years to find temporary jobs, delaying their marriages (Brown, 1993). This migration might not be needed for men who have access to inheritance.

The benefits in fertility could also result from higher chances of polygyny or serial monogamy for wealthy men. In an additional analysis (see details in the Supplementary Materials, Tables S15 and S16), we find that both Dogon men and women who have been married to a cousin at least once have more partners in their life than men and women who were only married to unrelated partners (2.4 partners on average for cousin-married men vs. 2.1 for unrelated-married men, expected difference for men: 0.29, *p* = 1; 1.8 partners on average for cousin-married women vs. 1.6 for unrelated-married women, expected difference for women: 0.22, *p* = 1). Our data do not allow us to distinguish between simultaneous and sequential partners. However, polygyny in Dogon is facilitated by land acquisition and increases men's reproductive success (Strassmann, 2003). In this society, polygyny is driven by an excess of women relative to men on the marriage market (Strassmann, 2000), which limits opportunities for women to choose a monogamous partner or to remarry. These imbalances might shape sexual conflicts in the context of cousin marriage. Men might use the inheritance they gain by marrying a cousin to attract other wives, possibly unrelated ones. Women married to cousins might also experience the fitness costs that may be connected to polygyny in this population (Strassmann, 2017).

Our population projection results provide some support for the *sexual conflict hypothesis*. The projected growth rate is lower for lineages of women practising cousin marriage than for lineages of women not practising cousin marriage. In contrast, lineages of men practising cousin marriage have similar growth rates to lineages of men not practising it. These asymmetries in fitness between men and women in the Dogon population might be paired with asymmetries also within families, between siblings, and between parents and children. The potential sexual conflict we detected in both populations is often also associated with family conflicts (Lessells, 2012), because it can lead to competition between siblings over resources, parental investment and marriage partners. Since the number of cousins in a family is limited, and polygyny in the Dogon is strictly non-sororal (Strassmann & Kurapati, 2016), when a Dogon woman marries a cousin, her sisters will need to find a mate somewhere else, and this task can take some time. Moreover, marriage exchanges in the Dogon happen more often between villages than between families (Brown, 1993), so it is unlikely that a cousin marriage of one sibling implies increased chances of marriage for the other siblings. Asymmetries in the age at first reproduction between those subpopulations practising cousin marriage and those not practising it might be consistent with this scenario.

We were limited in assessing whether cousin marriage might be associated with a conflict between parents and offspring over marriage arrangements. As cousin marriages in the Dogon are mostly arranged by parents (Strassmann, 2003), we could expect that parents make arrangements that provide themselves with fitness benefits. In an additional analysis (see details in the Supplementary Materials, Tables S13 and S14), we find that both women and men who have at least one child married to a cousin have more grandchildren than women and men whose children only married unrelated partners, even when accounting for the number of children they themselves have (for a given number of children, individuals with a child married to a cousin have on average about twice as many grandchildren; for example, men with five children have on average 1.02 more grandchildren per child, *p* = 1, and women on average have 1.30 more grandchildren per child, *p* = 1). These results could be connected to the aforementioned sibling competition in families practising cousin marriage: children (especially sons) who marry a cousin could compensate with their higher fertility for the potentially lower fertility of their siblings. However, we cannot draw definitive conclusions on whether this indeed represents parent–offspring conflict. We do not know whether parents make marriage arrangements that are optimal for each of their children given the available partners. Moreover, these additional models only focus on fertility, while our analyses indicated that cousin-married individuals differ in other important parameters (e.g. offspring survival rates) from unrelated-married individuals. Further research would be required to specifically investigate the potential fitness asymmetries that are expected under parent-offspring conflict.

Overall, hypothetical lineages – composed of women and men born to and married to cousins – that represent families practising cousin marriage have slightly lower growth rates than families not practising cousin marriage. This reduced overall fitness is likely to be a consequence of sexual conflict (Rankin et al., 2011) and fitness costs that are not fully compensated for by benefits. It is unclear if cousin marriage might have previously been adaptive in the Dogon. It is hard to draw conclusions from current data, because changes in the social and marriage systems of the Dogon arising from Islamization might have affected the costs and benefits of cousin marriage.

#### 4.2.2. Ancien Régime

In the Ancien Régime nobility, the life-history consequences of cousin marriage are less striking: differences between lineages practising cousin marriage and those not practising it are more limited than what we observe in the Dogon. The genetic load of the population could be lower because of the lower overall frequency of marriages between relatives (Charlesworth & Willis, 2009) or the more benign environment that a high social status may provide. In such contexts, inbreeding costs may not be as visible. The benefits of cousin marriage in this population are more limited too: other marriage strategies, such as marriage exchanges with unrelated dynasties abroad, might give equal benefits in terms of mate access without generating costs.

Yet, among the Ancien Régime nobility, we can detect some differences in key demographic characteristics. Here, fitness costs are mainly carried by men and fitness benefits are mainly gained by women. Sons born to parents who are cousins are less likely to survive to reproductive age than sons born to unrelated parents. This difference is not visible for daughters. The biological effects of mating with kin might have had an impact on the survival of boys more than on the survival of girls, because of a change in the causes of infant deaths (from external to perinatal), that particularly affected the more vulnerable male offspring during this period (Drevenstedt et al., 2008). Alternatively, the lower survival of male offspring in families practising cousin marriage could be due to differential parental investment. Families that practice cousin marriage and strict marriage arrangements might also practice primogeniture and invest less in younger sons (Hrdy & Judge, 1993).

Women married to cousins show similar fertility, and an earlier age at first reproduction than women who marry unrelated partners. Men married to cousins show slightly lower total fertility than men married to unrelated partners. Even by remarrying, men seem not to completely offset the fertility cost of cousin marriage. An additional analysis (see details in the Supplementary Materials, Tables S31 and S32) shows that men married to cousins have a slightly higher chance of remarriage than men married to unrelated partners, whereas women married to cousins have a similar chance of remarriage as women married to unrelated partners (1.74 partners on average for cousin-married men vs. 1.43 for unrelated-married men, expected difference for men: 0.31, *p* = 0.96; 1.14 partners on average for cousin-married women vs. 1.07 for unrelated-married women, expected difference for women: 0.07, *p* = 0.68). Our demographic approach does account for the total number of children men have with all of their wives though. We cannot exclude the possibility that men might offset fertility costs through illegitimate children: the rate of illegitimate births in European countries in the covered period ranged from 1 to 6% (Brée, 2014; Muir, 2018). However, it is probably just as likely that men married to unrelated partners had illegitimate children at similar rates.

As for the benefits of cousin marriage, in a society where monogamy is the rule, and marrying a non-noble individual is not an option (Hurwich, 1998), marrying a cousin could make the task of finding a partner easier for women (as predicted by the *mate scarcity hypothesis*). In this population, the problem of finding a mate is in fact related to social structure. Among the nobility, marriages were under parental control (Tulchin, 2013), and cousin marriages in particular were mostly arranged by parents (Goody, 1983). Marriages of daughters were costly under a dowry system (Goody, 1983). The possibility of avoiding dowry payments and keeping resources in the family could act as an incentive for noble parents to arrange marriages within the family, rather than with unrelated dynasties. This selection pressure could, in turn, lead to lower ages at first marriage if cousins are scarce. Since cousin marriage allowed parents to marry more daughters, and intermarriages among related families could be frequent (Alvarez et al., 2009), we can speculate that, at a family level, cousin marriage could reduce sibling competition, especially among sisters. Interestingly, unlike women, men married to cousins do not show earlier ages at first reproduction compared with their counterparts, while men born to cousins do show earlier ages at first reproduction compared with men born to unrelated parents. This discrepancy in age at first marriage between ‘married to cousin’ and ‘born to cousin’ groups is not well understood, but may reflect secular changes not well captured by our model.

Parent–offspring conflict might also be linked to the patterns of cousin marriage among the Ancien Régime. In an additional analysis (see details in the Supplementary Materials, Tables S29 and S30), we observe that both men and women who have at least one child married to a cousin also have more grandchildren than individuals who do not have children married to a cousin (for a given number of children, individuals with a child married to a cousin have on average about 1.2 as many grandchildren; for example, men with five children have on average 0.76 more grandchildren per child than men who do not have any child married to a cousin (*p* = 1) and who have the same number of children; women who have at least one child married to a cousin have an average of 0.67 more grandchildren per child than women who do not have any child married to a cousin (*p* = 1) and who have the same number of children). This suggests that, among the Ancien Régime nobility, parents might benefit from arranging a cousin marriage by having more of their children married. However, the same caveats mentioned above for the Dogon also apply here, and analyses that specifically target asymmetries in fitness within and between families would be needed to study this topic in more detail.

### 4.3. The potential advantages and limitations of demographic approaches to understanding the prevalence of cousin marriage

One limitation of our empirical approach is that it requires individuals to be classified into discrete populations for the purposes of estimating survival and fertility functions. Such classifications are obviously oversimplifications, and individuals can change marriage status over time. In order to gain some degree of empirical tractability, we classified individuals as cousin-married if they had been married to a cousin at least once (regardless of their other marriages). Therefore, the offspring counts of a small subset of multiply-married individuals may include children sired by unrelated partners. Further research would be needed to determine if cousin-married individuals compensate for inbreeding costs by subsequently marrying unrelated individuals.

An additional problem with classifying individuals into groups solely on the basis of marriage type, is that this approach does not reveal the underlying fitness trade-offs within families (Chagnon et al., 2017). Our supplemental analyses, however, give some insights into the costs and benefits for parents and siblings of cousin-married couples. It would be interesting to further investigate possible asymmetries in fitness between same-sex and different-sex siblings, especially in regards to the chances of (cousin) marriage. Moreover, more work is needed to determine the preferred marriage strategies of the siblings of cousin-married individuals.

Furthermore, the simplistic population-level demographic approaches that we use here are only designed to estimate simple differences in projected population growth rate as a function of marriage system. These models, however, can be extended to link the potential drivers of cousin marriage (e.g. inheritance and wealth considerations) to survival and fertility functions, and then to resulting lineage growth rate differences. Such approaches would be complementary to those that use individual-level information directly (Johow et al., 2019; Shenk et al., 2016).

Another question we did not address is whether cousin marriage may be a bet-hedging strategy, where, despite producing a lower growth rate over short intervals of time, cousin marriage carries adaptive benefits in terms of reduced variance in fitness in the long run. Such a bet-hedging strategy could be selected for because it increases geometric mean fitness across generations (Starrfelt & Kokko, 2012). This could be relevant in the Dogon case, where periodic droughts and other environmental changes can have rare but extreme effects on fertility and survival (Brown, 1993). A stochastic population projection model, where rates of survival and fertility are allowed to change at each time interval, could address this issue (e.g. as in Puleston et al., 2014). Such an empirical analysis, however, would require even longer-term samples than those we relied on here.

Empirical estimation of demographic effects requires systematic data, in terms of both breadth (complete or at least systematic sampling of individuals) and depth (long temporal periods of observation). Inferences from limited, non-systematically collected samples are likely to be non-representative. Comparative datasets, which include both cousin-married and non-cousin-married individuals and deploy whole-population censuses over multiple generations are rare. In this respect, the Dogon and Ancien Régime databases are some of the best available data sources. Nevertheless, the low sample size of individuals born or married to cousins limits our ability to draw hard conclusions about the potential adaptiveness of cousin marriage. Another issue impacting our analyses is that there are some missing birth and death dates for individuals who died in infancy, especially in the Ancien Régime database (see Methods). Since our survival and fertility estimation procedures are age-specific, we may therefore underestimate fertility and overestimate childhood survival rates.

## 5. Conclusion

Despite the aforementioned limitations, the analyses presented here show that population projection approaches, which account for between-marriage-system differences in survival and fertility across the whole life-span, can usefully represent the adaptive consequences of cousin marriage. Our analyses indicate possible trade-offs between fitness costs and benefits in connection to cousin marriage, and give insights into the mechanisms shaping these trade-offs in two human populations. We find evidence of inbreeding costs – mainly in offspring survival during childhood – in both sexes, in both populations. We also find some benefits of cousin marriage, mainly in fertility and age at first reproduction. Our results are consistent with the *kin benefits hypothesis* and the *mate scarcity hypothesis*. Owing to data limitations, we did not directly investigate the possible origins of these benefits, but our methods can in principle be extended to measure the relationship between survival, fertility and various covariate measures, like material wealth.

Our findings also give insights into how the potential benefits of cousin marriage are linked to specific socio-ecological conditions. We find that fertility benefits are mainly gained by men in the Dogon and by women in the Ancien Régime nobility. This indicates that, in these two populations, men and women are affected differently by cousin marriage, and thus possibly have different motivations for pursuing the strategy. Dogon men, who live in a society permitting polygyny which has various rules for male inheritance, can use cousin marriage to successfully compete in a difficult marriage market. By encouraging cousin marriage, Dogon lineages can preserve inherited land holdings, minimize out-migration of men and facilitate earlier marriages and even polygamous unions. In contrast, women in the Ancien Régime, who live in a monogamous society characterized by dowry payments, can use cousin marriage to minimize the costs of marriages. Analyses at the population-level reveal asymmetries in fitness between the sexes and between marriage-type groups. Age-structured models give a multifaceted picture of fitness costs and benefits. Distinguishing between survival in childhood and adulthood has helped us disentangle inbreeding costs and possible fitness benefits. The combined analysis of key life history traits, along with assessment of the long-term dynamics implied by differences in projected growth rates for lineages practising different marriage strategies, gives insight into whether a strategy with potential fitness costs – e.g. cousin marriage – can nevertheless serve an adaptive function. More broadly, given the increasing availability of genealogical data from various human populations, demographic analyses like those presented here can provide insights into the origins and maintenance of various behaviours.

## Data Availability

The datasets and the R and Stan code for reproducing the results can be accessed on the Open Science Framework: https://doi.org/10.17605/OSF.IO/DGUF5. The original datasets of Dogon (Boni) and Ancien Régime are available from the Kinsources archive: https://www.kinsources.net.

## Supplementary material

For supplementary material accompanying this paper visit https://doi.org/10.1017/ehs.2022.55. click here to view supplementary material
