# Supplementary material for: Fitness consequences of cousin marriage: a life-history assessment in two populations
Source: Evol Hum Sci. 2022 Nov 29;5:e3. doi: 10.1017/ehs.2022.55 (PMC10426087; doi:10.1017/ehs.2022.55)
Supplement: Supplementary file 1 [file S2513843X2200055Xsup001.pdf]

Supplementary Materials for: "Fitness consequences of cousin marriage: a life-history assessment in two populations".

Arianna Dalzero, Cody T. Ross, Dieter Lukas

November 2022

Contents

1 Tables S1-S32 showing the posterior estimates of the results and of the differences (contrasts) in the life history estimates between sets of sub-populations. 2

2 Figures S1-S5 illustrating the posterior estimates of the age-specific demographic moments and of the growth rates and sensitivities. 20

1 Tables S1-S32 showing the posterior estimates of the results and of the differences (contrasts) in the life history estimates between sets of sub-populations.

Table S1: Dogon: child survival results.

marriage	sex	state	mean	low PI	up PI
Cousin	Male	Born	0.63	0.60	0.66
Cousin	Female	Born	0.65	0.62	0.69
Unrelated	Male	Born	0.68	0.66	0.69
Unrelated	Female	Born	0.72	0.70	0.73

Table 1: Mean (fourth column), 5% lower (fifth column) and 94% upper boundary (sixth column) of the 89% percentile interval of the posterior probability distributions of the Dogon sub-populations, male and female (second column), married and born (third column) in families practicing cousin marriage or not (first column), for survival up to age 12. For child survival we report only the results for offspring sub-populations (born) because all the individuals who are married survived up to reproductive age.

Table S2: Dogon: child survival contrasts.

sex	state	mean	low PI	up PI	P>0
Male	Born	-0.04	-0.08	-0.01	0.02
Female	Born	-0.06	-0.10	-0.03	0.00

Table 2: Mean (third column), 5% lower (fourth column) and 94% upper boundary (fifth column) of the 89% percentile interval and fraction above 0 (sixth column) of the posterior probability distributions for the contrasts (subtraction difference) between the posterior probability distributions of Dogon sub-populations, male and female (first column), born (second column) in families practicing cousin marriage versus not practicing it (e.g., male individuals born to cousins versus male individuals born to unrelated parents), for child survival. For child survival we report only the contrasts for offspring sub-populations (born), because all the individuals who are married survived up to reproductive age.

Table S3: Dogon: adult survival results.

marriage	sex	state	mean	low PI	up PI
Cousin	Male	Born	0.66	0.58	0.74
Cousin	Female	Born	0.64	0.57	0.72
Unrelated	Male	Born	0.66	0.63	0.69
Unrelated	Female	Born	0.75	0.72	0.77
Cousin	Male	Married	0.72	0.66	0.77
Cousin	Female	Married	0.76	0.70	0.81
Unrelated	Male	Married	0.71	0.68	0.75
Unrelated	Female	Married	0.76	0.73	0.78

Table 3: Mean (fourth column), 5% lower (fifth column) and 94% upper boundary (sixth column) of the 89% percentile interval of the posterior probability distributions of the Dogon sub-populations, male and female (second column), married and born (third column) in families practicing cousin marriage or not (first column), for survival from age 12 to 60.

16

Table S4: Dogon: adult survival contrasts.

sex	state	mean	low PI	up PI	P>0
Male	Born	0.02	-0.12	0.17	0.59
Female	Born	-0.10	-0.17	-0.03	0.01
Male	Married	0.04	-0.06	0.15	0.75
Female	Married	-0.01	-0.06	0.04	0.37

Table 4: Mean (third column), 5% lower (fourth column) and 94% upper boundary (fifth column) of the 89% percentile interval and fraction above 0 (sixth column) of the posterior probability distributions for the contrasts (subtraction difference) between the posterior probability distributions of Dogon sub-populations, male and female (first column), born and married (second column) in families practicing cousin marriage versus not practicing it (e.g., male individuals born to cousins versus male individuals born to unrelated parents), for survival from age 12 to 60.

17

Table S5: Dogon: fertility results

marriage	sex	state	mean	low PI	up PI
Cousin	Male	Born	4.36	4.00	4.73
Cousin	Female	Born	3.13	2.87	3.39
Unrelated	Male	Born	4.08	3.96	4.20
Unrelated	Female	Born	3.30	3.21	3.40
Cousin	Male	Married	4.41	4.17	4.65
Cousin	Female	Married	3.29	3.10	3.48
Unrelated	Male	Married	4.01	3.88	4.15
Unrelated	Female	Married	3.29	3.19	3.39

Table 5: Mean (fourth column), 5% lower (fifth column) and 94% upper boundary (sixth column) of the 89% percentile interval of the posterior probability distributions of the Dogon sub-populations, male and female (second column), married and born (third column) in families practicing cousin marriage or not (first column), for cumulative fertility (cumulative sum) in reproductive ages. These values refer to the probability of giving birth to a same-sex child (number of female children for women and male children of men).

18

Table S6: Dogon: fertility contrasts.

sex	state	mean	low PI	up PI	P>0
Male	Born	0.28	-0.09	0.67	0.88
Female	Born	-0.17	-0.44	0.10	0.16
Male	Married	0.39	0.12	0.66	0.99
Female	Married	-0.00	-0.21	0.21	0.48

Table 6: Mean (third column), 5% lower (fourth column) and 94% upper boundary (fifth column) of the 89% percentile interval and fraction above 0 (sixth column) of the posterior probability distributions for the contrasts (subtraction difference) between the posterior probability distributions of Dogon sub-populations male and female (first column), born and married (second column) in families practicing cousin marriage versus not practicing (e.g., male individuals married to cousins versus male individuals married to unrelated parents) for cumulative fertility (cumulative sum) in all the reproductive ages. These values refer to the probability of giving birth to a same-sex child (number of female children for women and male children of men).

19

Table S7: Dogon: fertility contrasts born versus married.

sex	marriage	mean	low PI	up PI	P>0
Male	Cousin	-0.05	-0.48	0.39	0.43
Female	Cousin	-0.16	-0.47	0.16	0.21
Male	Unrelated	0.06	-0.12	0.24	0.71
Female	Unrelated	0.01	-0.13	0.14	0.55

Table 7: Mean (third column), 5% lower (fourth column) and 94% upper boundary (fifth column) of the 89% percentile interval and fraction above 0 (sixth column) of the posterior probability distributions for the contrasts (subtraction difference) between the posterior probability distributions of Dogon sub-populations male and female (first column), practicing cousin marriage and not practicing it (second column), born versus married (e.g., male individuals born to cousins versus male individuals married to cousins) for cumulative fertility (cumulative sum) in reproductive ages. These values refer to the probability of giving birth to a same-sex child (number of female children for women and male children of men). These additional comparisons have not been presented in the manuscript.

20

Table S8: Dogon: age at first reproduction results.

marriage	sex	state	mean	low PI	up PI
Cousin	Male	Born	27.01	26.00	28.00
Cousin	Female	Born	21.15	20.00	22.00
Unrelated	Male	Born	26.41	26.00	27.00
Unrelated	Female	Born	20.99	21.00	21.00
Cousin	Male	Married	25.99	26.00	26.00
Cousin	Female	Married	20.12	20.00	21.00
Unrelated	Male	Married	26.95	27.00	27.00
Unrelated	Female	Married	21.00	21.00	21.00

Table 8: Mean (fourth column), 5% lower (fifth column) and 94% upper boundary (sixth column) of the 89% percentile interval of the posterior probability distributions of the Dogon sub-populations, male and female (second column), married and born (third column) in families practicing cousin marriage or not (first column), for age at first reproduction. Age at first reproduction is defined as the first age when the cumulative probability of fertility (probability to give birth to a same sex child) exceeds 0.5.

21

Table S9: Dogon: age at first reproduction contrasts.

sex	state	mean	low PI	up PI	P>0
Male	Born	0.60	-1.00	2.00	0.57
Female	Born	0.15	-1.00	1.00	0.22
Male	Married	-0.95	-1.00	0.00	0.00
Female	Married	-0.88	-1.00	0.00	0.00

Table 9: Mean (third column), 5% lower (fourth column) and 94% upper boundary (fifth column) of the 89% percentile interval and fraction above 0 (sixth column) of the posterior probability distributions for the contrasts (subtraction difference) between the posterior probability distributions of Dogon sub-populations male and female (first column), born and married (second column) in families practicing cousin marriage versus not practicing (e.g., male individuals married to cousins versus male individuals married to unrelated parents), for age at first reproduction. Age at first reproduction is defined as the first age when the cumulative probability of fertility (probability to give birth to a same sex child) exceeds 0.5.

22

Table S10: Dogon: age at first reproduction contrasts born versus married.

sex	marriage	mean	low PI	up PI	P>0
Male	Cousin	1.01	0.00	2.00	0.81
Female	Cousin	1.02	0.00	2.00	0.84
Male	Unrelated	-0.54	-1.00	0.00	0.02
Female	Unrelated	-0.01	0.00	0.00	0.00

Table 10: Mean (third column), 5% lower (fourth column) and 94% upper boundary (fifth column) of the 89% percentile interval and fraction above 0 (sixth column) of the posterior probability distributions for the contrasts (subtraction difference) between the posterior probability distributions of Dogon sub-populations male and female (first column), practicing cousin marriage and not practicing it (second column), born versus married (e.g., male individuals born to cousins versus male individuals married to cousins), for age at first reproduction. Age at first reproduction is defined as the first age when the cumulative probability of fertility (probability to give birth to a same sex child) exceeds 0.5. These additional comparisons have not been presented in the manuscript.

23

Table S11: Dogon: growth rate results.

marriage	sex	state	mean	low PI	up PI
Cousin	Male	Born	1.0247	1.0218	1.0276
Cousin	Female	Born	1.0208	1.0172	1.0244
Unrelated	Male	Born	1.0260	1.0249	1.0271
Unrelated	Female	Born	1.0286	1.0273	1.0298
Cousin	Two sex	Born	1.0249	1.0221	1.0276
Unrelated	Two sex	Born	1.0249	1.0221	1.0276
Cousin	Male	Married	1.0414	1.0397	1.0431
Cousin	Female	Married	1.0426	1.0403	1.0449
Unrelated	Male	Married	1.0391	1.0380	1.0400
Unrelated	Female	Married	1.0415	1.0404	1.0426
Cousin	Two sex	Married	1.0429	1.0411	1.0449
Unrelated	Two sex	Married	1.0415	1.0404	1.0426
Cousin	Men	Born/married	1.0256	1.0232	1.0281
Cousin	Women	Born/married	1.0238	1.0208	1.0268
Unrelated	Men	Born/married	1.0254	1.0243	1.0266
Unrelated	Women	Born/married	1.0282	1.0269	1.0295
Cousin	Two sex	Born/married	1.0260	1.0239	1.0282
Unrelated	Two sex	Born/married	1.0282	1.0269	1.0295

Table 11: Mean (fourth column), 5% lower (fifth column) and 94% upper boundary (sixth column) of the 89% percentile interval of the posterior probability distributions of the Dogon hypothetical populations, male, female and two-sex (second column), born, married and mixed born and married (third column) in lineages practicing cousin marriage or not (first column), for projected growth rate. The growth rates of “married” hypothetical populations have not been presented in the manuscript. The growth rate is the real part of the dominant eigenvalue of the Leslie matrix.

24

Table S12: Dogon: growth rate contrasts.

sex	state	mean	low PI	up PI	P>0
Male	Born	-0.0012	-0.0044	0.0018	0.2620
Female	Born	-0.0078	-0.0116	-0.0040	0.0003
Two sex	Born	-0.0037	-0.0067	-0.0007	0.0252
Male	Married	0.0023	0.0004	0.0043	0.9698
Female	Married	0.0011	-0.0015	0.0037	0.7483
Two sex	Married	0.0014	-0.0007	0.0037	0.8403
Male	Born/married	0.0002	-0.0025	0.0029	0.5468
Female	Born/married	-0.0043	-0.0077	-0.0011	0.0163
Two sex	Born/married	-0.0022	-0.0048	0.0003	0.0797

Table 12: Mean (third column), 5% lower (fourth column) and 94% upper boundary (fifth column) of the 89% percentile interval and fraction above 0 (sixth column) of the posterior probability distributions for the contrasts (subtraction difference) between the posterior probability distributions of Dogon hypothetical populations, male, female and two-sex (first column), born, married and mixed born and married (second column) in lineages practicing cousin marriage versus not practicing (e.g., female lineages practicing cousin marriage versus female lineages not practicing cousin marriage), for the projected growth rate. Results of comparisons between “married” populations have not been presented in the manuscript. The growth rate is the real part of the dominant eigenvalue of the Leslie matrix.

Table S13: Dogon: grandchildren results.

child marriage	sex	mean	low PI	up PI
Cousin	Male	11.97	11.41	12.51
Unrelated	Male	6.88	6.52	7.26
Cousin	Female	16.71	16.20	17.23
Unrelated	Female	10.24	9.91	10.57

Table 13: Mean (third column), 5% lower (fourth column) and 94% upper boundary (fifth column) of the 89% percentile interval of the posterior probability distributions of the Dogon individuals, male and female (second column), with at least a child married to a cousin or without any child married to a cousin (first column), for number of grandchildren. Individuals with a child married to a cousin are the grandparents of individuals who are born to cousins and individuals without any child married to a cousin are grandparents only of individuals born to unrelated parents. These estimates have been extracted from a Poisson model with number of grandchildren as response variable and cousin marriage of at least one child (intercept), and number of children as explanatory variables (with an interaction between explanatory variables, see additional analyses). These estimates refer to the number of grandchildren of individuals who have 5 children.

26

Table S14: Dogon: grandchildren contrasts.

sex	mean	low PI	up PI	P>0
Male	1.02	0.88	1.16	1
Female	1.30	1.17	1.42	1

Table 14: Mean (second column), 5% lower (third column) and 94% upper boundary (fourth column) of the 89% percentile interval and fraction above 0 (fifth column) of the posterior probability distributions for the contrasts (subtraction difference) between the posterior probability distributions of Dogon individuals, male and female (first column), with at least one child married to a cousin versus without any child married to a cousin (e.g., male individuals with at least a child married to a cousin vs male individuals without any child married to a cousin), for number of grandchildren (controlling for number of children, see Table S13 and additional analyses). The contrast is scaled by the number of children we are predicting for (5) and refers to the difference in number of grandchildren per each child. To translate these absolute contrasts into relative terms, averaging across the number of children, we built a separate model, with number of children as intercept. This additional model indicates that, for a given number of children, men with a child married to a cousin are expected to have 2.14 (89% percentile interval 1.76 - 2.58) as many grandchildren as men with children only married to unrelated partners, and women 1.84 (89% percentile interval 1.43-2.32) as many grandchildren as women with children only married to unrelated partners.

27

Table S15: Dogon: partners results.

marriage	sex	mean	low PI	up PI
Cousin	Male	2.39	2.26	2.51
Unrelated	Male	2.10	2.04	2.16
Cousin	Female	1.77	1.67	1.88
Unrelated	Female	1.55	1.50	1.60

Table 15: Mean (third column), 5% lower (fourth column) and 94% upper boundary (fifth column) of the 89% percentile interval of the posterior probability distributions of the Dogon individuals, male and female (second column), married to a cousin or married to unrelated partner (first column), for number of partners in the life time. These estimates have been extracted from a Poisson model with number of partners as response variable and type of marriage as explanatory variable (see additional analyses).

Table S16: Dogon: partners contrasts.

sex	mean	low PI	up PI	P>0
Male	0.29	0.15	0.43	1
Female	0.22	0.11	0.34	1

Table 16: Mean (second column), 5% lower (third column) and 94% upper boundary (fourth column) of the 89% percentile interval and fraction above 0 (fifth column) of the posterior probability distributions for the contrasts (subtraction difference) between the posterior probability distributions of Dogon individuals, male and female (first column), married to a cousin versus married to an unrelated partner (e.g., male individuals married to a cousin vs male individuals married to unrelated partners), for number of partners (see Table S15 and additional analyses).

Table S17: Ancien Régime: child survival results.

marriage	sex	state	mean	low PI	up PI
Cousin	Male	Born	0.76	0.69	0.82
Cousin	Female	Born	0.84	0.77	0.89
Unrelated	Male	Born	0.82	0.80	0.83
Unrelated	Female	Born	0.85	0.83	0.86

Table 17: Mean (fourth column), 5% lower (fifth column) and 94% upper boundary (sixth column) of the 89% percentile interval of the posterior probability distributions of the Ancien Régime sub-populations, male and female (second column), born (third column) in families practicing cousin marriage or not (first column), for survival up to age 12. For child survival we report only the results for offspring sub-populations (born) because all the individuals who are married survived up to reproductive age.

Table S18: Ancien Régime: child survival contrasts.

sex	state	mean	low PI	up PI	P>0
Male	Born	-0.06	-0.13	0.01	0.09
Female	Born	-0.01	-0.07	0.05	0.42

Table 18: Mean (third column), 5% lower (fourth column) and 94% upper boundary (fifth column) of the 89% percentile interval and fraction above 0 (sixth column) of the posterior probability distributions for the contrasts (subtraction difference) between the posterior probability distributions of Ancien Régime sub-populations, male and female (first column), born and married (second column) in families practicing cousin marriage versus not practicing it (e.g., male individuals born to cousins vs male individuals born to unrelated parents), for child survival. For child survival we report only the contrasts for offspring sub-populations (born) because all the individuals who are married survived up to reproductive age.

Table S19: Ancien Régime: adult survival results.

marriage	sex	state	mean	low PI	up PI
Cousin	Male	Born	0.53	0.44	0.61
Cousin	Female	Born	0.43	0.34	0.51
Unrelated	Male	Born	0.49	0.47	0.52
Unrelated	Female	Born	0.49	0.47	0.52
Cousin	Male	Married	0.50	0.40	0.60
Cousin	Female	Married	0.46	0.36	0.56
Unrelated	Male	Married	0.51	0.47	0.54
Unrelated	Female	Married	0.47	0.43	0.51

Table 19: Mean (fourth column), 5% lower (fifth column) and 94% upper boundary (sixth column) of the 89% percentile interval of the posterior probability distributions of the Ancien Régime sub-populations, male and female (second column), married and born (third column) in families practicing cousin marriage or not (first column), for survival from age 12 to 60.

Table S20: Ancien Régime: adult survival contrasts.

sex	state	mean	low PI	up PI	P>0
Male	Born	0.03	-0.06	0.12	0.70
Female	Born	-0.07	-0.15	0.02	0.12
Male	Married	-0.01	-0.12	0.09	0.44
Female	Married	-0.01	-0.12	0.10	0.42

Table 20: Mean (third column), 5% lower (fourth column) and 94% upper boundary (fifth column) of the 89% percentile interval and fraction above 0 (sixth column) of the posterior probability distributions for the contrasts (subtraction difference) between the posterior probability distributions of Ancien Régime sub-populations, male and female (first column), born and married (second column) in families practicing cousin marriage versus not practicing it (e.g., male individuals born to cousins vs male individuals born to unrelated parents), for survival from age 12 to 60.

Table S21: Ancien Régime: fertility results.

marriage	sex	state	mean	low PI	up PI
Cousin	Male	Born	2.40	1.92	2.94
Cousin	Female	Born	2.24	1.79	2.72
Unrelated	Male	Born	2.22	2.10	2.34
Unrelated	Female	Born	2.31	2.18	2.44
Cousin	Male	Married	2.61	2.25	2.98
Cousin	Female	Married	2.59	2.18	3.02
Unrelated	Male	Married	2.82	2.67	2.97
Unrelated	Female	Married	2.61	2.48	2.76

Table 21: Mean (fourth column), 5% lower (fifth column) and 94% upper boundary (sixth column) of the 89% percentile interval of the posterior probability distributions of the Ancien Régime sub-populations, male and female (second column), married and born (third column) in families practicing cousin marriage or not (first column), for cumulative fertility (cumulative sum) in reproductive ages. These values refer to the probability of giving birth to a same-sex child (number of female children for women and male children of men).

Table S22: Ancien Régime: fertility contrasts.

sex	state	mean	low PI	up PI	P>0
Male	Born	0.18	-0.31	0.73	0.70
Female	Born	-0.06	-0.54	0.44	0.41
Male	Married	-0.21	-0.60	0.20	0.20
Female	Married	-0.02	-0.46	0.43	0.46

Table 22: Mean (third column), 5% lower (fourth column) and 94% upper boundary (fifth column) of the 89% percentile interval and fraction above 0 (sixth column) of the posterior probability distributions for the contrasts (subtraction difference) between the posterior probability distributions of Ancien Régime sub-populations male and female (first column), born and married (second column) in families practicing cousin marriage versus not practicing (e.g., male individuals married to cousins versus male individuals married to unrelated parents) for cumulative fertility (cumulative sum) in all the reproductive ages. These values refer to the probability of giving birth to a same-sex child (number of female children for women and male children of men).

Table S23: Ancien Régime: fertility contrasts born versus married.

sex	marriage	mean	low PI	up PI	P>0
Male	Cousin	-0.21	-0.83	0.44	0.29
Female	Cousin	-0.35	-0.98	0.28	0.19
Male	Unrelated	-0.60	-0.80	-0.41	0.00
Female	Unrelated	-0.31	-0.49	-0.12	0.00

Table 23: Mean (third column), 5% lower (fourth column) and 94% upper boundary (fifth column) of the 89% percentile interval and fraction above 0 (sixth column) of the posterior probability distributions for the contrasts (subtraction difference) between the posterior probability distributions of Ancien Régime sub-populations male and female (first column), practicing cousin marriage and not practicing it (second column), born versus married (e.g., male individuals born to cousins versus male individuals married to cousins) for cumulative fertility (cumulative sum) in reproductive ages. These values refer to the probability of giving birth to a same-sex child (number of female children for women and male children of men). These additional comparisons have not been presented in the manuscript.

Table S24: Ancien Régime: age at first reproduction results.

marriage	sex	state	mean	low PI	up PI
Cousin	Male	Born	24.36	21.00	27.00
Cousin	Female	Born	21.39	19.00	24.00
Unrelated	Male	Born	27.93	27.00	29.00
Unrelated	Female	Born	23.08	23.00	24.00
Cousin	Male	Married	26.25	25.00	28.00
Cousin	Female	Married	21.39	20.00	23.00
Unrelated	Male	Married	26.08	26.00	27.00
Unrelated	Female	Married	22.55	22.00	23.00

Table 24: Mean (fourth column), 5% lower (fifth column) and 94% upper boundary (sixth column) of the 89% percentile interval of the posterior probability distributions of the Ancien Régime sub-populations, male and female (second column), married and born (third column) in families practicing cousin marriage or not (first column), for age at first reproduction. Age at first reproduction is defined as the first age when the cumulative probability of fertility (probability to give birth to a same sex child) exceeds 0.5.

Table S25: Ancien Régime: age at first reproduction contrasts.

sex	state	mean	low PI	up PI	P>0
Male	Born	-3.58	-7.00	-1.00	0.01
Female	Born	-1.69	-5.00	1.00	0.09
Male	Married	0.17	-1.00	2.00	0.34
Female	Married	-1.17	-3.00	1.00	0.08

Table 25: Mean (third column), 5% lower (fourth column) and 94% upper boundary (fifth column) of the 89% percentile interval and fraction above 0 (sixth column) of the posterior probability distributions for the contrasts (subtraction difference) between the posterior probability distributions of Ancien Régime sub-populations male and female (first column), born and married (second column) in families practicing cousin marriage versus not practicing (e.g., male individuals married to cousins versus male individuals married to unrelated parents), for age at first reproduction. Age at first reproduction is defined as the first age when the cumulative probability of fertility (probability to give birth to a same sex child) exceeds 0.5.

Table S26: Ancien Régime: age at first reproduction born versus married contrasts.

sex	marriage	mean	low PI	up PI	P>0
Male	Cousin	-1.90	-5.00	1.00	0.13
Female	Cousin	0.00	-3.00	3.00	0.42
Male	Unrelated	1.85	1.00	3.00	0.99
Female	Unrelated	0.53	0.00	2.00	0.50

Table 26: Mean (third column), 5% lower (fourth column) and 94% upper boundary (fifth column) of the 89% percentile interval and fraction above 0 (sixth column) of the posterior probability distributions for the contrasts (subtraction difference) between the posterior probability distributions of Ancien Régime sub-populations male and female (first column), practicing cousin marriage and not practicing it (second column), born versus married (e.g., male individuals born to cousins versus male individuals married to cousins), for age at first reproduction. These additional comparisons have not been presented in the manuscript. Age at first reproduction is defined as the first age when the cumulative probability of fertility (probability to give birth to a same sex child) exceeds 0.5.

Table S27: Ancien Régime: growth rate results.

marriage	sex	state	mean	low PI	up PI
Cousin	Male	Born	1.0136	1.0055	1.0214
Cousin	Female	Born	1.0178	1.0084	1.0267
Unrelated	Male	Born	1.0138	1.0120	1.0155
Unrelated	Female	Born	1.0203	1.0181	1.0225
Cousin	Two sex	Born	1.0192	1.0118	1.0269
Unrelated	Two sex	Born	1.0192	1.0118	1.0269
Cousin	Male	Married	1.0257	1.0206	1.0306
Cousin	Female	Married	1.0301	1.0229	1.0367
Unrelated	Male	Married	1.0295	1.0277	1.0313
Unrelated	Female	Married	1.0318	1.0296	1.0341
Cousin	Two sex	Married	1.0308	1.0253	1.0368
Unrelated	Two sex	Married	1.0319	1.0299	1.0341
Cousin	Men	Born/married	1.0162	1.0105	1.0218
Cousin	Women	Born/married	1.0229	1.0153	1.0302
Unrelated	Men	Born/married	1.0214	1.0195	1.0232
Unrelated	Women	Born/married	1.0252	1.0230	1.0274
Cousin	Two sex	Born/married	1.0233	1.0170	1.0302
Unrelated	Two sex	Born/married	1.0252	1.0230	1.0274

Table 27: Mean (fourth column), 5% lower (fifth column) and 94% upper boundary (sixth column) of the 89% percentile interval of the posterior probability distributions of the Ancien Régime hypothetical populations, male, female and two-sex (second column), born, married and mixed born and married (third column) in lineages practicing cousin marriage or not (first column), for projected growth rate. The growth rates of “married” hypothetical populations have not been presented in the manuscript. The growth rate is the real part of the dominant eigenvalue of the Leslie matrix.

Table S28: Ancien Régime: growth rate contrasts.

sex	state	mean	low PI	up PI	P>0
Male	Born	-0.0002	-0.0083	0.0079	0.4860
Female	Born	-0.0025	-0.0124	0.0069	0.3417
Two sex	Born	-0.0012	-0.0089	0.0070	0.3988
Male	Married	-0.0038	-0.0093	0.0014	0.1280
Female	Married	-0.0017	-0.0091	0.0054	0.3545
Two sex	Married	-0.0011	-0.0070	0.0053	0.3667
Male	Born/married	-0.0051	-0.0111	0.0009	0.0860
Female	Born/married	-0.0023	-0.0102	0.0052	0.3193
Two sex	Born/married	-0.0019	-0.0086	0.0052	0.3223

Table 28: Mean (third column), 5% lower (fourth column) and 94% upper boundary (fifth column) of the 89% percentile interval and fraction above 0 (sixth column) of the posterior probability distributions for the contrasts (subtraction difference) between the posterior probability distributions of Ancien Régime hypothetical populations, male, female and two-sex (first column), born, married and mixed born and married (second column) in lineages practicing cousin marriage versus not practicing (e.g., female lineages practicing cousin marriage versus female lineages not practicing cousin marriage), for the projected growth rate. Results of comparisons between "married" populations have not been presented in the manuscript. The growth rate is the real part of the dominant eigenvalue of the Leslie matrix.

Table S29: Ancien Régime: grandchildren results.

child marriage	sex	mean	low PI	up PI
Cousin	Male	12.11	11.35	12.91
Unrelated	Male	8.28	8.02	8.53
Cousin	Female	12.01	11.24	12.80
Unrelated	Female	8.68	8.37	8.98

Table 29: Mean (third column), 5% lower (fourth column) and 94% upper boundary (fifth column) of the 89% percentile interval of the posterior probability distributions of the Ancien Régime individuals, male and female (second column), with at least a child married to a cousin or not (first column), for number of grandchildren. Individuals with a child married to a cousin are the grandparents of individuals who are born to cousins and individuals without any child married to a cousin are grandparents only of individuals born to unrelated parents. These estimates have been extracted from a Poisson model with number of grandchildren as response variable and cousin marriage of at least one child (intercept) and number of children as explanatory variables (with an interaction between explanatory variables, see additional analyses). These estimates refer to the number of grandchildren of individuals who have 5 children.

42

Table S30: Ancien Régime: grandchildren contrasts.

sex	mean	low PI	up PI	P>0
Male	0.76	0.61	0.93	1
Female	0.67	0.50	0.84	1

Table 30: Mean (second column), 5% lower (third column) and 94% upper boundary (fourth column) of the 89% percentile interval and fraction above 0 (fifth column) of the posterior probability distributions for the contrasts (subtraction difference) between the posterior probability distributions of Dogon individuals, male and female (first column), with at least one child married to a cousin versus without any child married to a cousin (e.g., male individuals with at least a child married to a cousin vs male individuals without any child married to a cousin), for number of grandchildren (controlling for number of children, see Table S29 and additional analyses). The contrast is scaled by the number of children we are predicting for (5) and refers to the difference in number of grandchildren per each child. To translate these absolute contrasts into relative terms, averaging across the number of children, we also built a separate model, with number of children as intercept. This additional model indicates that, for a given number of children, men with a child married to a cousin are expected to have 1.18 (89% percentile interval 0.95-1.44) as many grandchildren as men with children only married to unrelated partners, and women 1.13 (89% percentile interval 0.89-1.41) as many grandchildren as men with children only married to unrelated partners.

43

Table S31: Ancien Régime: partners results.

marriage	sex	mean	low PI	up PI
Cousin	Male	1.73	1.46	2.02
Unrelated	Male	1.43	1.35	1.51
Cousin	Female	1.14	0.92	1.37
Unrelated	Female	1.07	1	1.13

Table 31: Mean (third column), 5% lower (fourth column) and 94% upper boundary (fifth column) of the 89% percentile interval of the posterior probability distributions of the Ancien Régime individuals, male and female (second column), married to a cousin or married to unrelated partner (first column), for number of partners in the life time. These estimates have been extracted from a Poisson model with number of partners as response variable and type of marriage as explanatory variable (see additional analyses).

Table S32: Ancien Régime: partners contrasts.

sex	mean	low PI	up PI	P>0
Male	0.30	0.02	0.60	0.96
Female	0.07	-0.15	0.31	0.68

Table 32: Mean (second column), 5% lower (third column) and 94% upper boundary (fourth column) of the 89% percentile interval and fraction above 0 (fifth column) of the posterior probability distributions for the contrasts (subtraction difference) between the posterior probability distributions of Ancien Régime individuals, male and female (first column), married to a cousin versus married to unrelated partner (e.g., male individuals married to a cousin vs male individuals married to unrelated partners), for number of partners (see additional analyses).

2 Figures S1-S5 illustrating the posterior estimates of the age-specific demographic moments and of the growth rates and sensitivities.

Figure S1: Dogon. Fertility of individuals born to cousins and to unrelated parents.

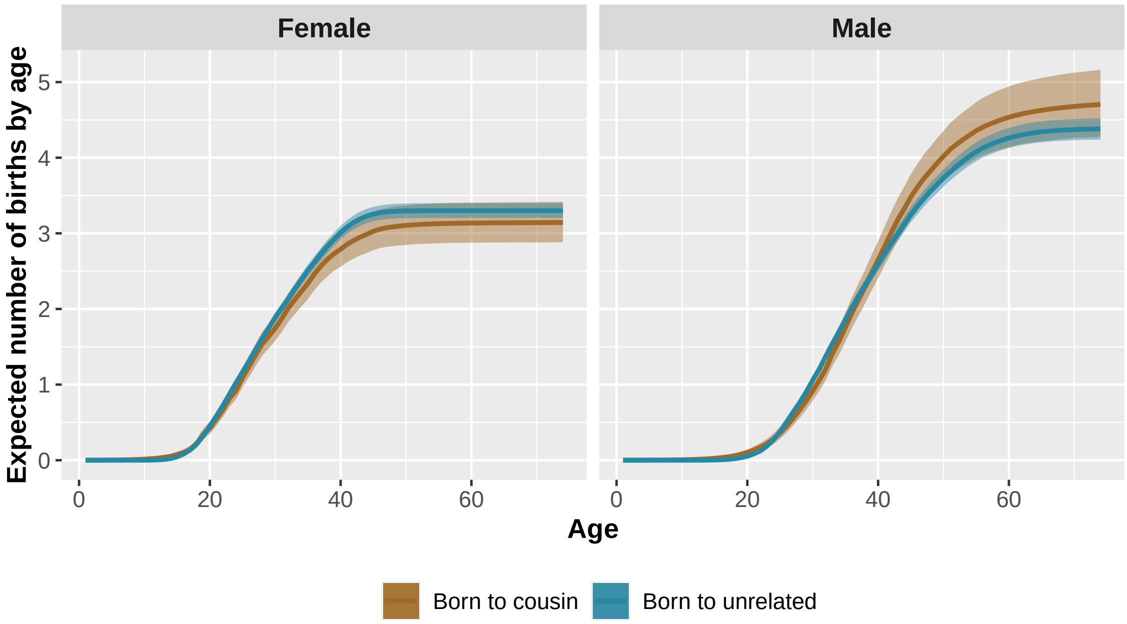

Figure 1: Expected number of children (y axis) for each age of life (x axis) for Dogon women (left panel) and men (right panel) who are born to cousins (brown) and born to unrelated parents (blue). These curves reflect the expected number of same-sex children (daughters of women and sons of men) produced by individuals.

Figure S2: Ancien Régime. Fertility of individuals born to cousins and to unrelated parents.

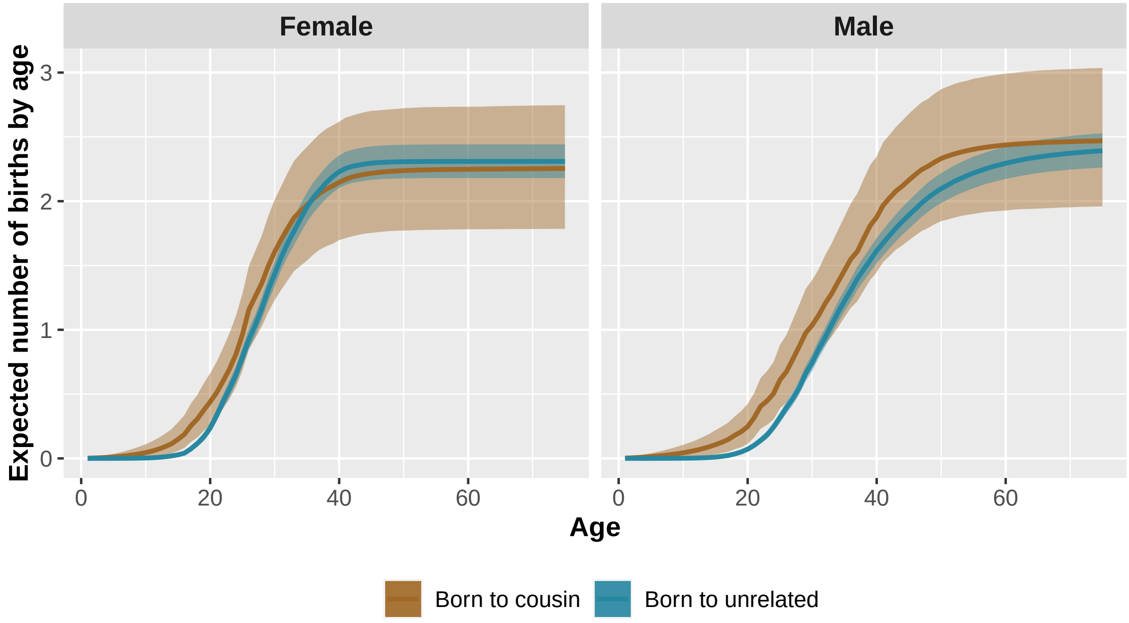

Figure 2: Expected number of children (y axis) for each age of life (x axis) for Ancien Régime women (left panel) and men (right panel) who are born to cousins (brown) and born to unrelated partners (blue). These curves reflect the expected number of same-sex children (daughters of women and sons of men) produced by individuals.

50

51 Figure S3: Dogon. Growth rates of sub-populations of individuals who are born to cousins and born to unrelated parents.

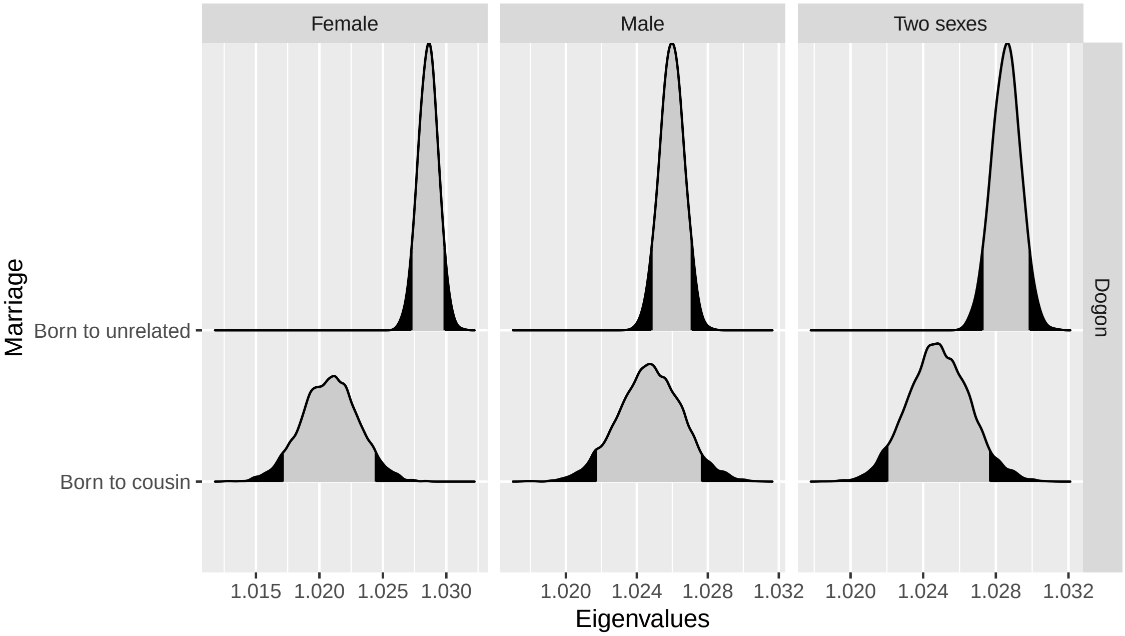

Figure 3: Projected growth rates shown here by the density of the posterior distribution of the estimated Eigenvalue (x axes), of Dogon hypothetical sub-populations of women (left column), men (central column), and men and women together (right column), from the Leslie matrices where we define parameters on the basis of birth. Survival and fertility rates are from individuals who are born to cousins (or born to unrelated parents). The growth rate of women and of women and men in lineages practicing cousin marriage (second row) is lower than the growth rate of women and women and men in lineages not practicing cousin marriage (first row). This suggests fitness costs for offspring of families practicing cousin marriage.

52

Figure S4: Ancien Régime. Growth rates of sub-populations of individuals who are born to cousins and born to unrelated parents.

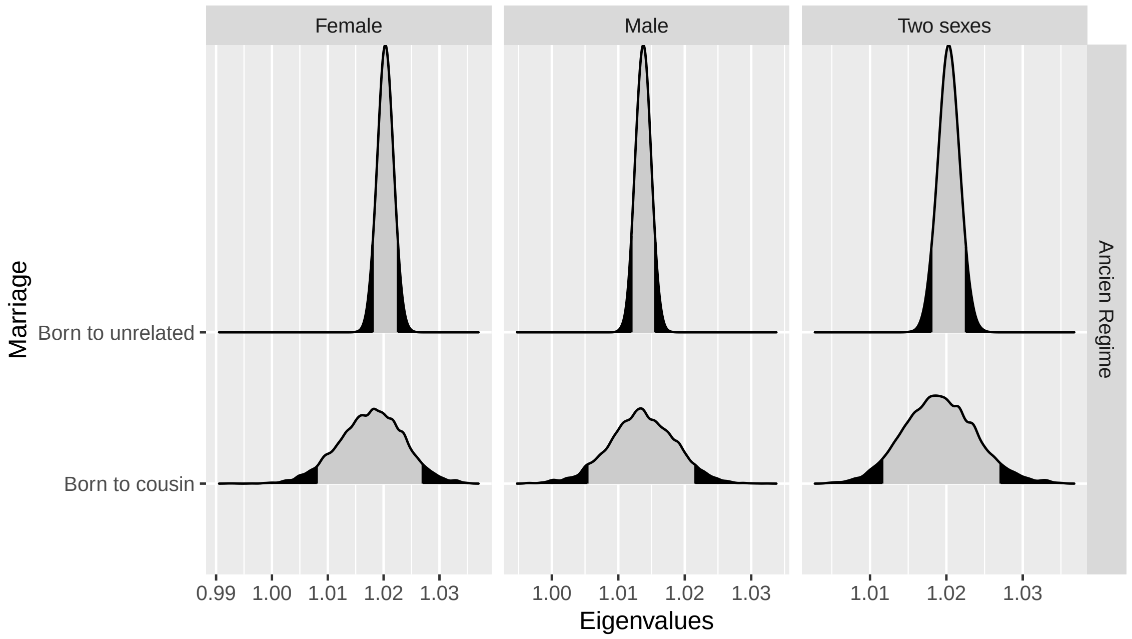

Figure 4: Projected growth rates shown here by the density of the posterior distribution of the estimated Eigenvalue (x axes), of Ancien Régime hypothetical sub-populations of women (left column), men (central column), and men and women together (right column), from the Leslie matrices where we define parameters on the basis of birth. Survival and fertility rates are from individuals who are born to cousins (or born to unrelated parents). There are no differences between lineages practicing cousin marriage (second row) and not practicing it (first row).

Figure S5: Dogon and Ancien Régime. Survival sensitivities.

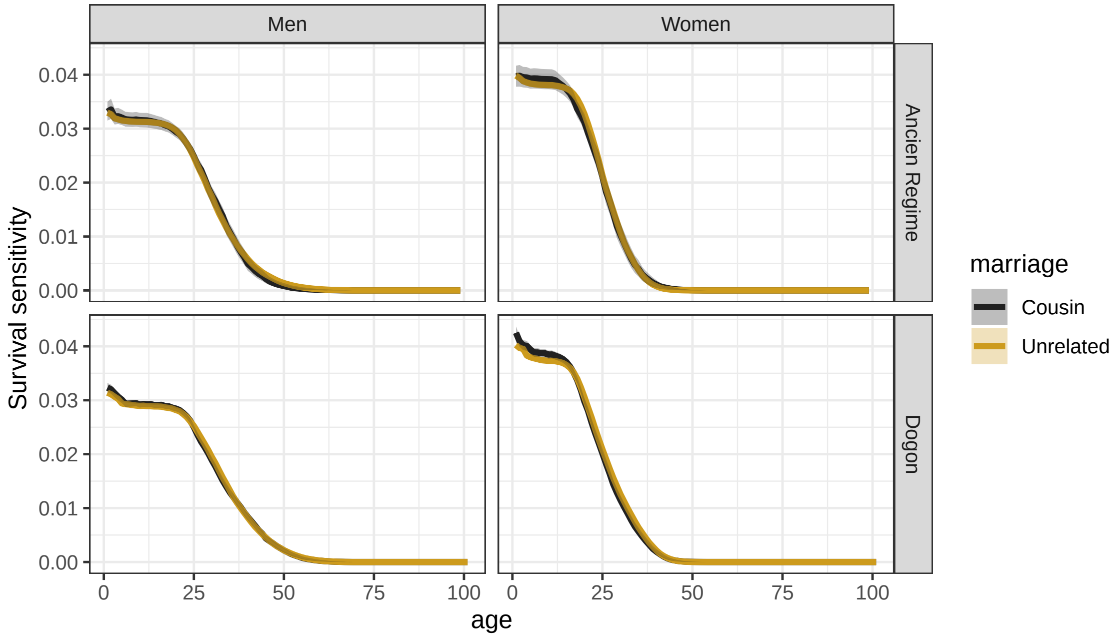

Figure 5: Sensitivity of the growth rate to changes in survival (y axes) for each reproductive age (x axes) of Dogon (bottom row) and Ancien Régime (upper row) hypothetical sub-populations of men (left column), and women (right column) practicing cousin marriage (black) and not practicing it (yellow). These sensitivities are from the Leslie matrices where we define parameters on the basis of birth and marriage. The shaded region represents the 90% quantile. In the both the Dogon and Ancien Régime, the survival sensitivity of the sub-population of women practicing cousin marriage is higher in early ages than the survival sensitivity of women not practicing it, whereas there are no differences between these groups in adult ages. The survival sensitivities of Dogon and Ancien Régime men practicing cousin marriage and not practicing it overlap in all ages.
